# proTRAC - a software for probabilistic piRNA cluster detection, visualization and analysis

**DOI:** 10.1186/1471-2105-13-5

**Published:** 2012-01-10

**Authors:** David Rosenkranz, Hans Zischler

**Affiliations:** 1 Institute of Anthropology, Johannes Gutenberg-University Mainz, Colonel-Kleinmann-Weg 2, 55099 Mainz, Germany

## Abstract

**Background:**

Throughout the metazoan lineage, typically gonadal expressed Piwi proteins and their guiding piRNAs (~26-32nt in length) form a protective mechanism of RNA interference directed against the propagation of transposable elements (TEs). Most piRNAs are generated from genomic piRNA clusters. Annotation of experimentally obtained piRNAs from small RNA/cDNA-libraries and detection of genomic piRNA clusters are crucial for a thorough understanding of the still enigmatic piRNA pathway, especially in an evolutionary context. Currently, detection of piRNA clusters relies on bioinformatics rather than detection and sequencing of primary piRNA cluster transcripts and the stringency of the methods applied in different studies differs considerably. Additionally, not all important piRNA cluster characteristics were taken into account during bioinformatic processing. Depending on the applied method this can lead to: i) an accidentally underrepresentation of TE related piRNAs, ii) overlook duplicated clusters harboring few or no single-copy loci and iii) false positive annotation of clusters that are in fact just accumulations of multi-copy loci corresponding to frequently mapped reads, but are not transcribed to piRNA precursors.

**Results:**

We developed a software which detects and analyses piRNA clusters (proTRAC, probabilistic TRacking and Analysis of Clusters) based on quantifiable deviations from a hypothetical uniform distribution regarding the decisive piRNA cluster characteristics. We used piRNA sequences from human, macaque, mouse and rat to identify piRNA clusters in the respective species with proTRAC and compared the obtained results with piRNA cluster annotation from piRNABank and the results generated by different hitherto applied methods.

proTRAC identified clusters not annotated at piRNABank and rejected annotated clusters based on the absence of important features like strand asymmetry. We further show, that proTRAC detects clusters that are passed over if a minimum number of single-copy piRNA loci are required and that proTRAC assigns more sequence reads per cluster since it does not preclude frequently mapped reads from the analysis.

**Conclusions:**

With proTRAC we provide a reliable tool for detection, visualization and analysis of piRNA clusters. Detected clusters are well supported by comprehensible probabilistic parameters and retain a maximum amount of information, thus overcoming the present conflict of sensitivity and specificity in piRNA cluster detection.

## Background

In a wide variety of animals, mainly germline expressed small RNAs - named Piwi interacting (pi)RNAs because of their interaction with effector Piwi proteins - play an important role as guiding RNAs in safeguarding the genome from the detrimental effects of actively transposing elements [1]. Most piRNAs are encoded in strand specific genomic clusters ranging from <1kb to >100kb. Beside mono-directional clusters encoding piRNAs on only one strand, there are also bi-directional clusters whose halves encode piRNAs on opposite strands and where transcription starts in opposite directions from a centrally located promoter. In general, piRNA clusters are assumed to be transcribed into long single stranded precursors that are subject to subsequent processing, leading to mature piRNAs. In a process referred to as ping pong cycle [2], piRNA guided Piwi proteins cleave TE transcripts thus producing a second population of TE derived piRNAs. Although piRNA genesis shows signs of a quasi-random mechanism with partially overlapping sequences, piRNAs exhibit typical sequence characteristics, e.g. position specific frequency patterns. In mice, the cluster derived piRNA population exhibits a strong bias for Uridine at the 5'-end, whereas the transposon derived population is biased for Adenine at position 10. In Drosophila, the situation is converse [3]. However, many questions concerning this process, as well as the functional role of piRNAs beyond transposon silencing (only 17% of mouse piRNAs correspond to TE sequences with the majority mapping only once to the genome [4]) remain elusive.

Research on piRNA biogenesis and function, as well as the successful targeting of questions related to the possible coevolution of the Piwi/piRNA system, will involve comparative studies of homologous piRNA clusters [5,6]. Therefore, a reliable bioinformatic piRNA cluster detection tool is imperative, especially in light of the ever exceeding amount of data obtained from next generation sequencing (NGS) that requires robust automated bioinformatic solutions.

Present studies identified piRNA clusters in the human, mouse and rat genome using different methods, starting with varying mismatch stringency when mapping the obtained sequence reads from piRNA transcriptome analyses to genomes. In addition, piRNA clusters were annotated at piRNABank [7] using the available data (table 1). The hitherto applied algorithms basically rely on finding regions that exhibit a high density of mapped piRNA sequences and respective threshold values depend on the amount of mapped sequences and are mostly determined in a heuristic manner, depending on whether the main focus lies on specificity or sensitivity. However, a considerable fraction of piRNA sequences, especially TE related sequences, also maps to regions in the genome that are most likely not transcribed to piRNA precursors, hence do not represent formal piRNA clusters. Purely by chance, these hits can accumulate e.g. in regions that exhibit a high amount of TEs and may accidentaly be annotated as piRNA cluster. In this context, stringent criteria such as used by Girard et al. 2006 [4] and Lau et al. 2006 [5] ensure high specificity by precluding sequence reads with high abundance or requiring a minimum number of single-copy piRNA loci respectively. On the other hand, they may lead to the exclusion of clusters that arose from recent duplication events [6] hence harboring no or only a few piRNA loci whose sequence is unique within the genome. Furthermore they may fail to identify all existent piRNA loci within a given cluster, when frequently mapped reads are a priori excluded from the analysis [4]. Less stringent criteria, relying solely on a high amount of piRNA loci such as used by Lakshmi and Agrawal 2007 [7], potentially increase sensitivity at the expense of specificity since frequently mapped sequence reads in particular can cumulatively map to regions in the genome that are not transcribed to piRNA precursors. Consequently, many of the annotated clusters at piRNABank indeed exhibit an appropriate number of putative piRNA loci within a small genomic region, but e.g. do not exhibit the mono- or bidirectional organization which is typical for piRNA clusters.

**Table 1:** Summary of piRNA cluster identification methods and results from previous studies

study	organism	identified clusters	criteria for detection of clusters
Aravin et al. 2006 [3]	human/mouse	14/42	at least 4 piRNA loci per cluster, maximum distance between two piRNA loci 15 kb

Girard et al. 2006 [4]	human/mouse/rat	186/123/157	at least 5 piRNA loci/5kb, at least 10 piRNA loci per cluster, only sequence reads that mapped 1-5 times to the genome were considered, sequence reads mapped to genome allowing up to 2 mismatches

Lau et al. 2006 [5]	mouse/rat	94/100	at least 20 single-copy loci, at least 1 piRNA locus/kb

Lakshmi and Agrawal 2007 [7]	human/mouse/rat	114/2710/189	at least n** single-copy loci/20kb, at least 2 piRNA loci/kb

Grivna et al. 2006 [12]	mouse	35	*

Watanabe et al. 2006 [13]	mouse	34	p < = 0.05, where p = (s/S)^n-1^×_N_C_n _S: genome size (bp), s: cluster size (bp), N: total number of sequence hits, n: number of hits in cluster

*method not explained in more detail, ** based on hit density for each organism [7].

Based on the imperfection of the currently available algorithms, and since the essential differences between them may hamper upcoming comparative studies in this field, we developed the user-friendly cluster detection software proTRAC, which uses SeqMap output files [8] for fast and customized detection, visualization and analysis of piRNA clusters in genomes, ensuring reproducibility and comparability. proTRAC analyzes the entirety of mapped sequence reads and identifies clusters based on significant deviations from the hypothetical uniform distribution regarding the density of mapped reads, strand asymmetry, frequency of putative piRNA loci with T at position 1 (1T) or A at position 10 (10A), as well as the amount of putative piRNA loci within the typical piRNA length range and the quantity of loci corresponding to infrequently mapped reads. The latter criterion represents a convenient benchmark since many piRNA loci sequences within one cluster exhibit low redundancy or are even unique within the genome. This causes an increased number of normalized hits within a piRNA cluster (normalized by the number of genomic hits produced by the respective sequence reads) as compared to the value that one would expect if a corresponding number of hits was randomly selected from the entirety of mapped reads. In the following we show, that the proTRAC algorithm provides considerable advantages compared to the currently applied methods.

## Implementation

### Workflow

**Figure 1:**
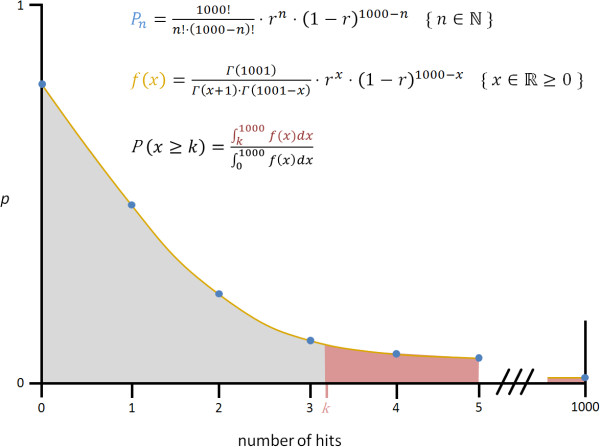
**Determining the minimum number of piRNA loci per kb**. *P*_*n *_(blue points) refers to the probability to observe *n *hits (*n*∈ℕ) per kb. *f(x) *(yellow line) defines the function which extends the probabilities to positive rational numbers. *P*(*x*≥*k*) is the probability to observe at least *k *hits per kb (*k∈ℝ*). r = number of putative piRNA loci/size of piRNA encoding region [nt].

Accepted input files are specially formatted SeqMap output files (ELAND3 format) which can be obtained by running SeqMap with the option/output_all_matches. ELAND3 files contain a list of mapped sequences and associated coordinates in genome coordinate order, each line corresponding to one mapped sequence read. proTRAC basically operates with a sliding window while reading the input file. The window size in lines is given by the required minimum number of mapped sequence reads per cluster. If the region encompassed by the first and last coordinate of the sliding window is small enough to exceed the minimum density of mapped reads (hit density), the respective loci are tagged. Contiguously tagged loci are assembled to cluster candidates being subject to a subsequent verification process. The required minimum number of putative piRNA loci (= mapped reads) and minimum hit density depend upon the provided dataset and the stated minimum score values, which are explained in more detail below. In order to determine the minimum hit density, proTRAC examines the distribution of mapped sequence reads for each chromosome or scaffold and specifically calculates a significant hit density by stepwise computation of the probability to observe an increasing number of hits within a 1kb window on the given chromosome or scaffold assuming a uniform distribution of mapped sequences (figure 1).

In order to obtain hit density threshold values with a resolution more precise than in steps of 1 hit/kb, proTRAC calculates probabilities (*P*) for non-integer hit numbers (*k∈ℝ*) per kb with an increment of 0.1 by using factorials deduced from the gamma function: *Γ*(*n*) = (*n*-1)!. The stepwise calculation of *P*(*x *≥ *k*) continues until the probability reaches the significance level. Then, the minimum hit density is defined as *k *hits/kb.

The minimum number of putative piRNA loci per piRNA cluster corresponds to the minimum number of loci needed to reach all stated score values (log_10 _of reciprocal probabilities) with the given dataset.

If necessary, since overlapping sections of proper hit density can result in one large cluster candidate which in sum falls below the minimum hit density, proTRAC performs a stepwise clipping of peripheral hits to find the largest possible cluster candidate. During a progressive process with an increasing number of hits, in each step all possible combinations of upstream and downstream hits are clipped from the cluster candidate ends and the effect on hit density is assessed for each combination. This process continues until a sufficient combination with the minimal number of hits is found and the hit density of the remaining cluster, comprising the highest possible number of putative piRNA loci, exceeds the required minimum. The removed hits, potentially forming a separate cluster, are assessed subsequently as a separate cluster candidate.

Since piRNA clusters are typically organized in a mono- or bidirectional manner proTRAC determines directionality by comparing the degree of mono- and bi-directionality. The degree of mono-directionality is simply given by the proportion of sequence reads encoded on the main strand (the strand which encodes the majority of mapped sequence reads). To determine the degree of bi-directionality, each cluster is split stepwise between every pair of hits that yields two fragments with each fragment comprising at least 25% of all hits or at least 10 hits respectively. Subsequently, the proportion of sequence reads encoded on the main strand is computed independently for each fragment and summed pro rata. The highest attainable value accounts for the degree of bi-directionality. If one or both values exceed the user defined directionality threshold, the cluster is assigned to the respective directionality category. Otherwise it is assessed to be non-directional.

Not to rely solely on tracing regions that exhibit a high hit density, and rather additionally consider the characteristics of the clustered putative piRNA loci, proTRAC now verifies each cluster candidate by examining its: i) number of normalized hits to total hits ratio, ii) extent of strand bias, iii) proportion of putative piRNA loci with 1T or 10A respectively and iv) proportion of putative piRNA loci within the typical length range. For each parameter proTRAC assigns score values based on a probabilistic scoring system which evaluates the probabilities to obtain the observed cluster characteristics in the light of the given dataset (e.g. a score value of 2.0 corresponds to a probability of 0.01). Regarding strand bias, we assume equal probabilities for one mapped sequence read to be encoded on either plus- or minus strand. Furthermore, we presume that the number of hits per cluster is very small compared to the total number of mapped sequence reads, so that proTRAC can calculate probabilities in a sampling-with-replacement fashion, which accelerates computation without a noticeable effect on the results.

**Figure 2:**
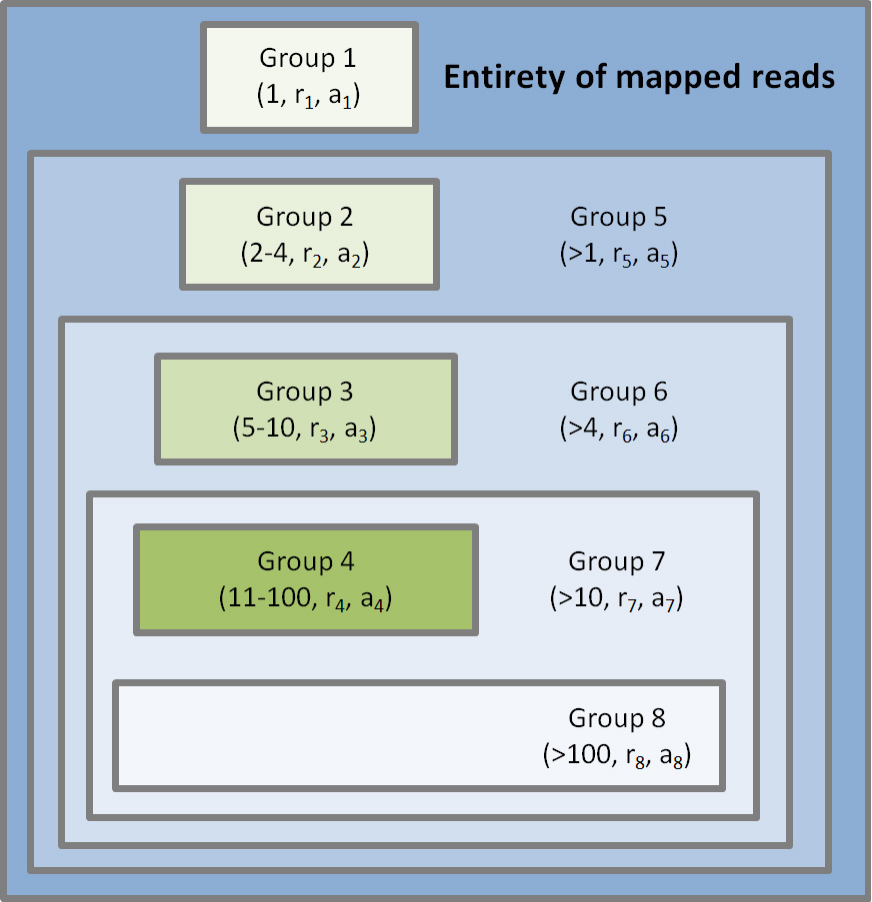
**Grouping of reads depending on the number of genomic hits they produce**. The grouping of sequence reads allows a fast approximated calculation of the NTR value. Data in brackets: hits per read, number of reads in group, average number of hits per read in this group.

Score values for strand bias (m = hits on main strand, r = 0.5), enrichment of putative piRNA loci with optimal length (m = putative piRNA loci with typical length in cluster, r = ratio of putative piRNA loci with typical length in dataset) and enrichment of putative piRNA loci with 1T or 10A (m = loci with 1T or 10A in cluster, r = ratio of putative piRNA loci with 1T or 10A in dataset) are calculated using the following equation: $score=\log{\left(\sum_{k=m}^{n}\left({}_{k}^{n}\right)\cdot r^{k}\cdot {\left(1-r\right)}^{n-k}\right)}^{-1}$ where *n *is the total number of hits in the cluster. The score value for enrichment of putative piRNA loci with 1T or 10A automatically scores either 1T or 10A enrichment (not the sum of both), so that it is suitable for datasets comprising primary as well as secondary piRNAs in both vertebrates and flies. For reasons of computational speed, proTRAC performs an approximate calculation of the score value for enrichment of putative piRNA loci corresponding to infrequently mapped reads as measured by the normalized-hits/total-hits ratio (NTR). Therefore, instead of considering the exact number of genomic hits produced by each read, which would lead to an exorbitant number of possible combinations per cluster, the entirety of mapped sequence reads (*R*) is sorted into 8 groups based on the number of genomic hits per read (figure 2). The number of groups was chosen as a suitable tradeoff between precision, which asymptotically increases, and computational speed, which exponentially decreases with a growing number of groups. For each of these groups containing r_1_-r_8 _reads, the average number of genomic hits per read is calculated (a_1_-a_8_) and this number is ascribed to each read of the group. For each cluster comprising *n *putative piRNA loci, proTRAC then calculates the minimum number of sequence reads from group 1 (*m*_*1*_), 2 (*m*_*2*_), 3 (*m*_*3*_) and 4 (*m*_*4*_) to obtain an NTR greater or equal than the observed NTR, under the assumption that all remaining reads are from group 5, 6, 7 and 8 respectively: $m_{i}=\left\lceil \frac{\frac{NTR-1}{a_{i+4}}\cdot n}{{\left(a_{i}\right)}^{-1}-{\left(a_{i+4}\right)}^{-1}}\right\rceil \;\{i\in \mathbb{N}|1,4\}$. Finally, proTRAC calculates the score based on the probability to obtain an NTR equal to or greater than the observed NTR by summating the probabilities for each sufficient combination: $score=\log{\left(\sum_{i=4}^{4}\sum_{k=m_{i}}^{n}\left({}_{k}^{n}\right)\cdot {\left(\frac{r_{i}}{R}\right)}^{k}\cdot {\left(\frac{r_{i+4}}{R}\right)}^{n-k}\right)}^{-1}$.

In general, the calculation of score values implies calculation of factorials corresponding to the total number of loci within one cluster, which gets computationally more expensive with an increasing number of hits per cluster candidate. If one cluster candidate comprises more than 1000 putative piRNA loci, its total number of putative piRNA loci is set to 1000 and the other parameters are scaled down accordingly. This may lead to an underestimation of score values. However, this will not lead to rejection of true clusters, since the computation of probability by default aborts once the probability falls below 1/10^100 ^(score = 100), which is often the case with clusters of this size. Moreover, the minimum set score values should reasonably not exceed 10.

Since it is possible, that real piRNA clusters are concealed by the presence of loci that correspond to frequently mapped sequence reads that do not originate from the cluster in question but distort its strand asymmetry, proTRAC optionally reevaluates rejected cluster candidates in that case, considering only loci that correspond to sequence reads that mapped not more than a stated maximum times to the genome, similar to the method applied by Girard et al. 2006 [4].

Once clusters are identified and validated, proTRAC calculates the probability for each cluster, whether any of its observed deviations from the hypothetical uniform distribution came off by chance and deduces the probability for 0, 1-or-more and 2-or-more mistakenly annotated clusters within the obtained set of detected clusters.

### Output

**Figure 3:**
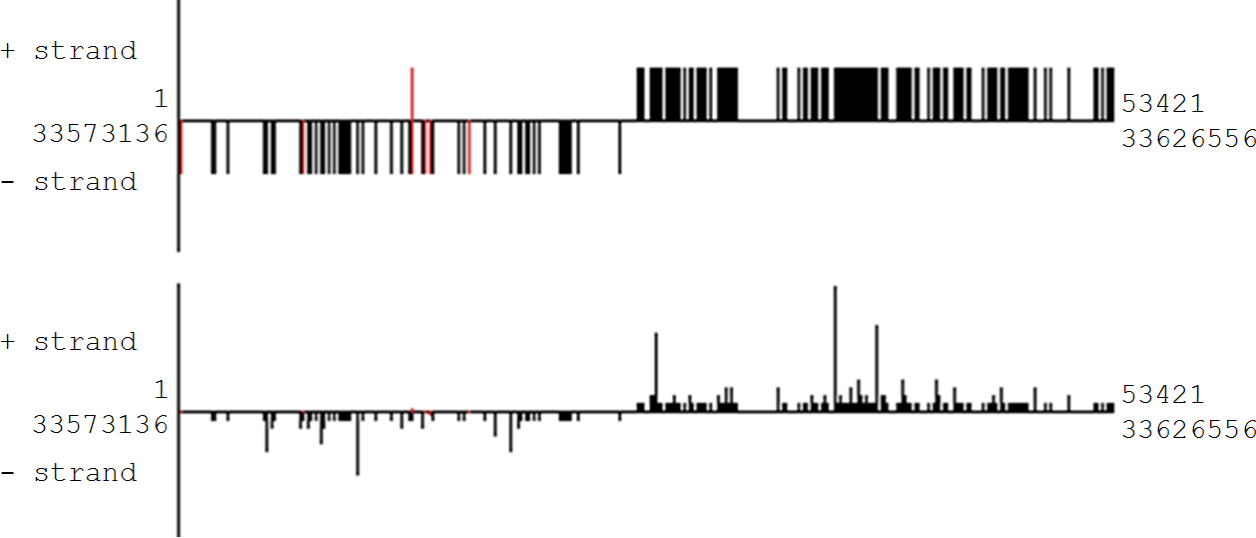
**Graphical proTRAC output**. Standard visualization (top) and customized visualization of the same cluster with indication of transcription rate and highlighting (red) of multi-copy loci (bottom). Sequence reads mapped to the plus-/minus-strand are directed upwards/downwards of the center line respectively. Upper coordinates refer to the length of the cluster, lower coordinates refer to the location of the cluster on the respective chromosome or scaffold.

If the initial sequence dataset that is mapped to the genome via SeqMap contains transcriptional information (FASTA titles refer to sequence read frequency), this information can be readout from the resulting ELAND3 file by proTRAC and used to display transcription rates of different putative piRNA loci within one cluster. In addition, loci corresponding to multiple mapping reads can be highlighted and their transcription rate can be normalized by the number genomic hits produced by the sequence read in question. Different piRNA cluster visualizations are displayed in figure 3.

Beside a separate FASTA file and picture file for each cluster, proTRAC can optionally output a summary file which lists all detected clusters with corresponding cluster data, optionally sorted by the summed score values.

## Results and Discussion

### Initial testing of program performance

In order to test the performance of proTRAC and to determine suitable thresholds for score values, nonredundant human (32046), mouse (34520) and rat (31357) piRNA sequences downloaded from National Center for Biotechnology Information (NCBI) nucleotide database [9] were mapped to the respective genomes (human NCBI Build37, mouse NCBI Build 37, rat RGSC3.4) via SeqMap (allowing only perfect matches, using the option/output_all_matches) and the SeqMap output files were used as input files for proTRAC.

proTRAC was performed three times for each dataset applying increasingly stringent thresholds. Assuming that the downloaded and mapped sequences contain piRNAs exclusively, we did not expect a deviation of putative piRNA loci with either T at position 1 or A at position 10 (1T/10A) in piRNA clusters as compared to the entirety of mapped sequences. Hence, calculation of the score value for enrichment of putative piRNA loci with 1T/10A was based on random base composition (25% for each nucleotide). The results are shown in table 2. The proTRAC summary files containing a list of all detected clusters with the related quality characteristics including the respective score values are available for each species (see additional files 1,2,3, piRNAclusters_human.txt, piRNAclusters_mouse.txt, piRNAclusters_rat.txt). Additionally, the respective zip-compressed proTRAC result folders containing a.png picture file and a FASTA sequence file for each cluster are available as additional files 4,5,6 (proTRAC_results_human.zip, proTRAC_results_mouse.zip, proTRAC results_rat.zip).

**Table 2:** Detection of human, mouse and rat piRNA clusters using proTRAC with different thresholds

species	thresholds^*^	minimum piRNA density [hits/kb]^**^	minimum loci/cluster	cluster candidates	tracked clusters (mono-, bi-, non-directional)^***^	p 0/≥2 mistakenly annotated clusters
	0.05/1.3	0.1 - 1.4	6	25402	368 (202, 120, 46)	0.001/0.992
human	0.01/2.0	0.6 - 2.4	8	14381	187 (139, 40, 8)	0.700/0.049
	0.001/3.0	1.5 - 3.5	11	4558	119 (99, 19, 1)	0.984/<0.001

	0.05/1.3	1.5 - 2.3	6	57260	242 (163, 63, 16)	0.133/0.595
mouse	0.01/2.0	2.5 - 3.4	8	51332	171 (129, 38, 4)	0.866/0.009
	0.001/3.0	3.7 - 4.7	11	37403	151 (123, 25, 3)	0.988/<0.001

	0.05/1.3	1.2 - 1.7	6	34069	186 (139, 43, 4)	0.334/0.293
rat	0.01/2.0	1.8 - 2.7	8	36402	168 (138, 28, 2)	0.926/0.003
	0.001/3.0	2.8 - 3.7	11	28576	162 (136, 26, 0)	0.994/<0.001

*Significance level for increased hit density/minimum score for strand bias, enrichment of loci corresponding to infrequently mapped reads and enrichment of putative piRNA loci with 1T/10A. **depending on chromosomal piRNA density. ***mono-, bi-directional: >75% of mapped sequences located on the main strand.

The inquiry of up to four different score values, corresponding to a particular probability, as well as the applied significance-based minimum hit density for each cluster, lead on to the statistical problem of multiple testing. According to the Bonferroni correction, we obtain the significance level α = 0.05 for the whole family of n (n = 5) tests by applying a significance level of 0.01 for each individual test (α/n). On this basis, we assume α = 0.01 (for hit density) and score values ≥2 to be suitable thresholds to yield results of adequate reliability.

For further assessment, we compared proTRAC results obtained with these thresholds with the clusters annotated at piRNABank as well as the results from Lau et al. 2006 [5]. Finally we repeated cluster detection with proTRAC taking only those sequence reads into account, that mapped 1-5 times to the genome, since only these sequence reads were considered by Girard et al. 2006 [4]. The resulting piRNA clusters were checked for the absence of loci that correspond to the excluded sequence reads.

### proTRAC results compared to piRNABank annotation

**Figure 4:**
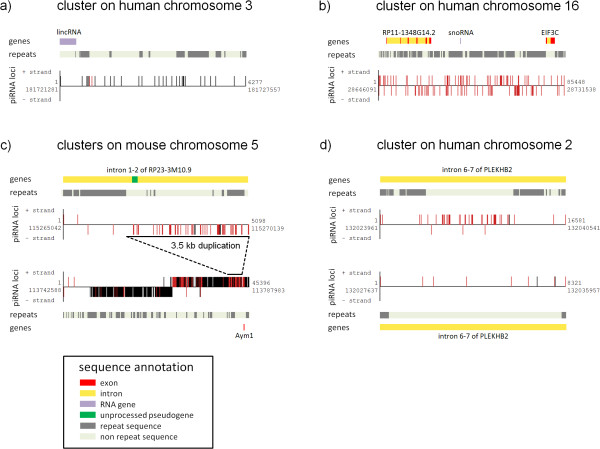
**Visualization of different piRNA clusters**. Every illustration of a piRNA cluster is associated with some sequence annotation which refers to the presence of repetitive sequences and genes. 4a: Cluster detected with proTRAC, not annotated at piRNABank. 4b: Cluster annotated at piRNABank, rejected by proTRAC because of its atypical topology. 4c: Upper illustration shows a cluster (not annotated by Lau et al. 2006 [5] but detected with proTRAC) arisen from a segmental duplication of the lower cluster. 4d: Two visualizations of the same cluster detected with different methods. Upper cluster (proTRAC) includes sequence reads that map more than 5 times to the genome, lower cluster (Girard et al. [4] method) does not include sequence reads that map more than 5 times to the genome. Black bars: single-copy loci, red bars: multi-copy loci.

proTRAC results apparently differ from piRNABank annotation. Beside a number of piRNA clusters that are present in both, proTRAC detects clusters not annotated in piRNABank and rejects other clusters annotated at piRNABank (table 3). In addition, some clusters detected with proTRAC are annotated as multiple clusters in piRNABank or vice versa. Running proTRAC without minimum probabilistic score values led to the detection of previously rejected clusters that are annotated at piRNABank. These clusters were found to exhibit characteristics casting doubts on their validity. Either, the putative piRNA loci do not show the typical mono- or bi-directionality and/or the NTR value does not significantly exceed the value that would be expected if a corresponding number of hits was randomly selected from the entirety of mapped sequence reads. Figure 4a and 4b examples show two human piRNA clusters, the first one detected with proTRAC and not annotated at piRNABank, the second one annotated at piRNABank and rejected by proTRAC. In addition, piRNABank annotation splits this cluster into two separate clusters (ID 85 and 86). The 172 hits within the second cluster (no single-copy locus) amount to only 5.6 normalized hits (proTRAC score for enrichment of putative piRNA loci corresponding to infrequently mapped sequence reads = 0.2) and therefore this cluster might simply constitute an accumulation of loci that correspond to frequently mapped reads that does not represent a formal piRNA cluster being transcribed to a piRNA precursor transcript. This finding is also supported by the atypical non-directional topology of the cluster (proTRAC score for strand bias = 0.2).

**Table 3:** Comparison of proTRAC results with piRNABank annotation

annotated by	human	rat	mouse
proTRAC and piRNABank*	84	84	91

only proTRAC	51	78	65

only piRNABank	27	102	2615

*if several clusters detected with proTRAC were annotated as one cluster in piRNABank or vice versa, these clusters were counted as one cluster.

### proTRAC results compared to results from Lau et al. 2006 [5]

The piRNA cluster criteria applied by Lau and colleagues (cf. table 1) are very stringent and suitable to mostly avoid false positive piRNA cluster annotation. However, they may lead to being insensitive regarding piRNA clusters that arose from recent paralogization events thus harboring no or only a few single-copy piRNA loci.

We searched for piRNA clusters applying the same sequence datasets as used by Lau and colleagues (piRNA sequences from Lau et al. 2006 [5] mapped to mouse build mm7 and rat build rn3). proTRAC confirmed all previously annotated clusters with the only exception of the X-chromosomal rat piRNA cluster 92 which undercuts the minimum required hit density of 2.7 hits/kb for the X chromosome (p ≤ 0.01). In addition, proTRAC detected 37 further mouse (total number of putative piRNA loci: 1255, normalized hits: 445, single-copy loci: 223) and 65 further rat (total number of putative piRNA loci: 6585, normalized hits: 1605, single-copy loci: 433) clusters. As an example, figure 4c shows two mouse piRNA clusters on chromosome 5, with the upper cluster arising from a 3.5kb duplication of the lower cluster. Although the duplicate harbors 175 putative piRNA loci (75.8 normalized hits), it is not annotated as piRNA cluster by Lau et al. [5] since it contains only 10 single-copy loci as a consequence of duplication.

### proTRAC results compared to results from Girard et al. 2006 [4]

In order to avoid false positive piRNA cluster annotation, Girard et al. [4] considered only sequence reads that mapped 1-5 times to the genome. We reproduced this method by running proTRAC taking only these infrequently mapped reads into account and applied a minimum hit density of 1 hits/kb and a minimum of 10 hits in total per piRNA cluster (cf. table 1) with the aim to assess the implications of rejecting those reads from the analysis. Excluding frequently mapped reads led to a decrease of the total number of detected clusters from 187 to 179, withal 47 of which were prior rejected because they lack a significant strand bias.

Regarding the assigned reads of the remaining 132 clusters identified with both methods, the total number of assigned reads that mapped once to the genome increased slightly from 12763 to 12836 which is caused by the lower hit density threshold (1 hits/kb) that led to an extension of piRNA cluster borders. However the number of assigned multiple mapping reads decreased drastically from 6560 to 3695. Excluding frequently mapped reads may also have a substantial influence on the resulting sequence composition of clustered piRNA loci since this will automatically exclude sequences that match to TEs with high copy number like LINEs or SINEs. In this way sequences that contribute to a major function of the Piwi-piRNA system, which is posttranscriptional silencing of actively transposing elements could be accidentally underrepresented.

Figure 4d shows a human piRNA cluster on chromosome 2, detected taking all sequence reads into account (top) and rejecting sequence reads that mapped more than 5 times to the genome (bottom). In the former case, 94 multiple mapping reads are assigned to this cluster, compared to 8 multiple mapping reads in the latter case.

### proTRAC performance in de novo piRNA cluster detection

**Figure 5:**
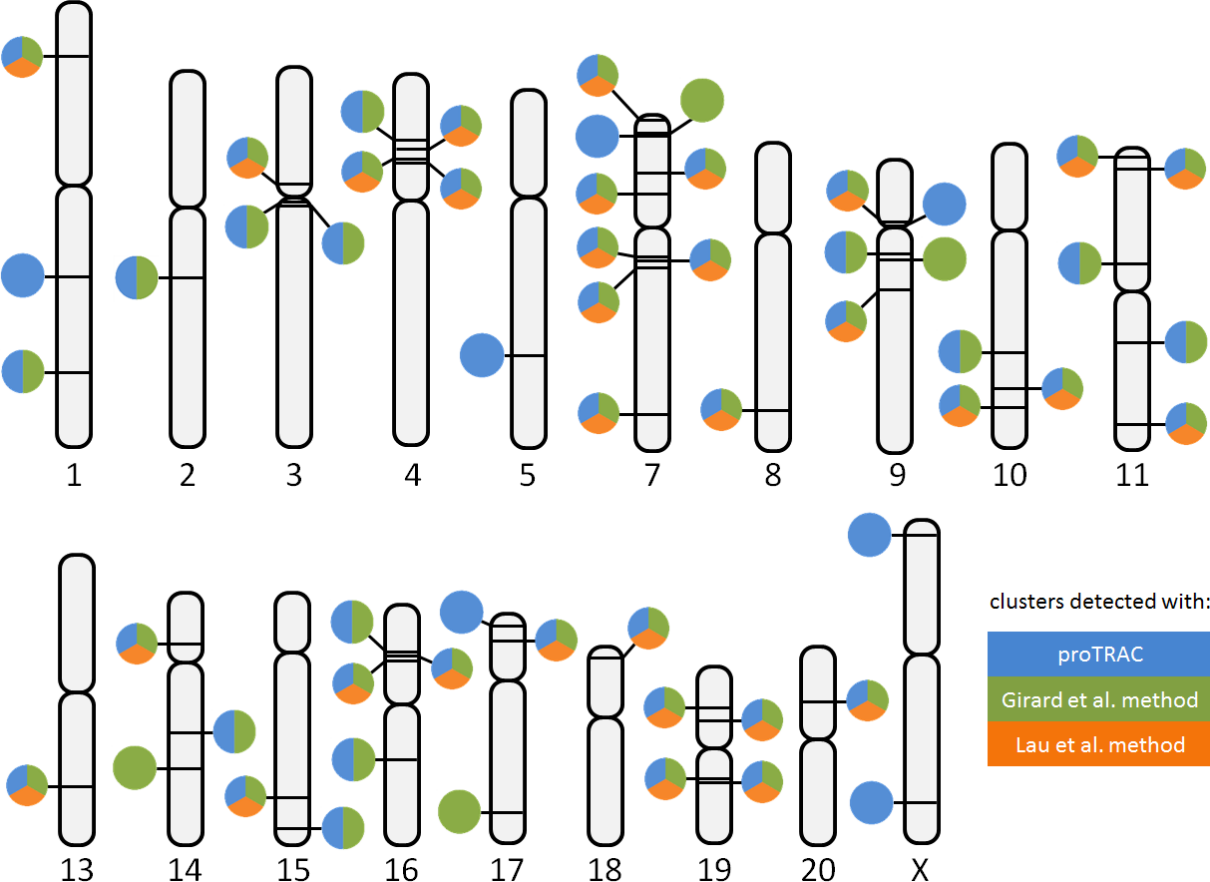
**Chromosomal distribution of piRNA clusters in *Macaca mulatta***. Bars on chromosomes indicate the locations of detected piRNA clusters. Each associated circle refers to the method with which this cluster could be detected (blue: proTRAC, green: method applied by Girard et al. [4], orange: method applied by Lau et al. [5]). Chromosome numbers are stated below the chromosomes. No clusters could be detected on chromosomes 6 and 11, as well as on the Y chromosome.

In order to evaluate the performance of proTRAC in de novo piRNA cluster detection in comparison to previously applied methods of cluster detection, we mapped piRNA like sequences obtained by 454-NGS of rhesus macaque testes RNA to the macaque genome (NCBI build 1.2) with SeqMap (6883 nonredundant sequences producing 452879 hits) and detected clusters with proTRAC and the methods applied by Girard et al. [4] and Lau et al. [5]. A detailed description of small RNA library preparation is available as additional file 7 (RNA_library_preparation.doc). The chromosomal distribution of the detected clusters is displayed in figure 5. 32 clusters could be detected using the method applied by Lau et al. [5]. Each of these clusters was also detected using either proTRAC, which detected 53 clusters comprising 5551 putative piRNA loci (4686 single-coply, 865 multi-copy) or the method applied by Girard et al. [4] which detected 49 detected clusters comprising 5240 piRNA loci (4687 single-copy, 553 multi-copy), albeit cluster borders and the number of assigned sequence reads can differ between methods. If several adjacent piRNA clusters detected with one method were identified as one cluster by any other method, these clusters were counted as one cluster.

Four small putative piRNA clusters (comprising 10 to 13 loci) were detected with the method from Girard et al. [4] but were rejected by proTRAC. Three of them because they lacked a significant strand bias, the forth because the respective putative piRNA loci did not show a significant enrichment for 1T or 10A. Seven clusters (with the largest one comprising 82 putative piRNA loci) were solely detected with proTRAC since each of them harbours less than ten loci that correspond to sequence reads producing 1-5 genomic hits. Nonetheless they exhibit all typical piRNA cluster characteristics. Figures and FASTA sequence files as well as a summary file of the detected piRNA clusters in rhesus macaque are available as additional file 8 (proTRAC_results_macaca.zip).

**Figure 6:**
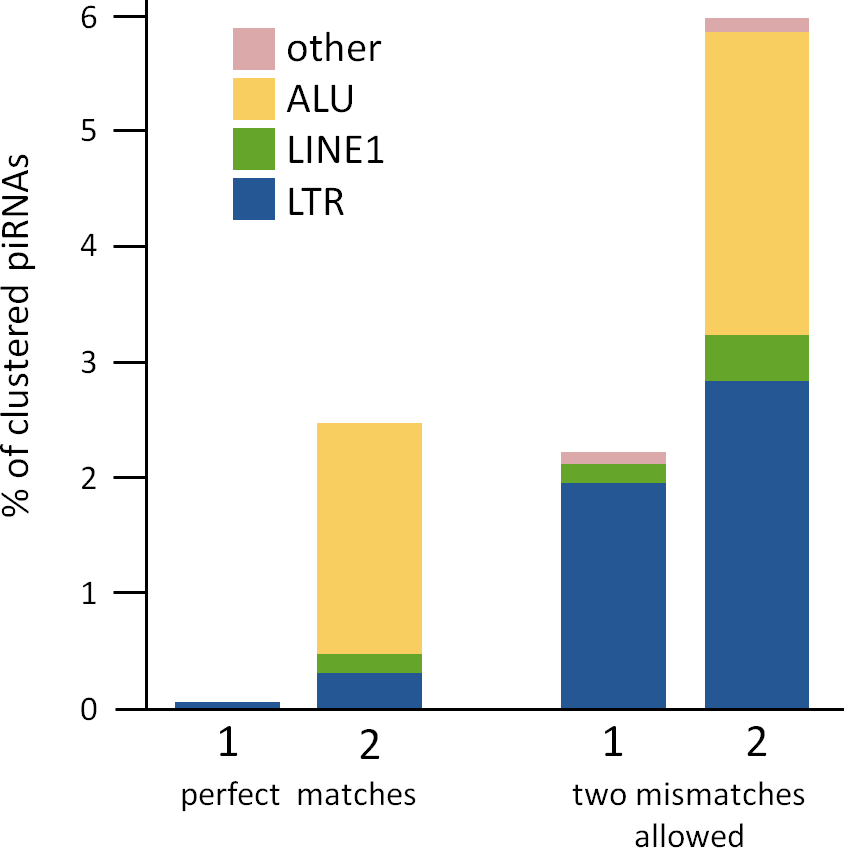
**Comparison of content and composition of TE related piRNAs in piRNA clusters detected with different methods**. Each bar itemizes the fraction of TE related and clustered sequence reads according to the different repeat classes. Bars labelled with 1 refer to putative piRNA clusters detected with the Girard et al. [4] method, bars labelled with 2 refer to putative piRNA clusters detected with proTRAC. Mapping piRNA like sequences to TE sequences was performed allowing only perfect matches (left side) and allowing up to two mismatches (right side).

In order to assess the effect of rejecting frequently mapped reads to sequence composition of piRNA clusters regarding the content of repeat matching piRNAs, clustered sequences were mapped to primate TE sequences downloaded from Repbase [10] (figure 6). Mapped sequence reads that match perfectly to TEs are virtually absent in clusters detected with the Girard et al. [4] method comprising 0.06% of all clustered sequence reads compared to 2.56% in clusters detected with proTRAC. The amount of TE matching sequence reads increased when we allowed up to two mismatches including insertions and deletions (2.27% and 5.96% respectively) but nevertheless is considerably lowered in clusters detected with the Girard et al. [4] method.

Furthermore, the sequence composition regarding different TE classes is apparently biased. While Alu related sequence reads constitute the major fraction of mapped sequence reads that match perfectly to TEs (~44% if two mismatches are allowed) in clusters detected with proTRAC, Alu matching sequence reads are completely absent and LTR matching sequence reads make up the vast majority of TE matching sequence reads in clusters detected with the Girard et al. [4] method. Caused by the extremely high copy number of Alu elements in primate genomes, being the most abundant mobile element of all [11], every Alu related and clustered sequence read from our dataset mapped more than five times to the macaque genome and hence was excluded from the dataset for cluster detection with the Girard et al. [4] method. Obviously, drawing conclusions in relation to specific piRNA functions from piRNA sequence composition relies on an unbiased representation of all existing piRNA loci in detected clusters which is one of the advantages of the proTRAC software. In summary, proTRAC detected more clusters than could be detected using the method applied by Lau et al. [5] and assigned more sequence reads to clusters than the method applied by Girard et al. [4] were rejection of frequently mapped reads led to an inadvertently underrepresentation as well as compositional bias of TE matching sequence reads.

## Conclusions

proTRAC provides a powerful tool for detection, visualization and analysis of piRNA clusters. Unlike hitherto applied methods for piRNA cluster identification, the proTRAC algorithm considers all hitherto described crucial piRNA cluster characteristics. Thus, piRNA clusters detected with proTRAC are well supported by comprehensible probabilistic parameters. In addition, proTRAC retains more information since it does not a priori preclude frequently mapped reads, which exclusively contribute to posttranscriptional transposon silencing, which was shown to lead to more assigned sequence reads per cluster in most cases and prevents accidentally underrepresentation and compositional bias of TE matching sequence reads. Moreover, proTRAC potentially allows clusters with only a few or even without single-copy loci, which leads to the detection of piRNA clusters arisen from segmental duplications that are passed over when using algorithms that require a fixed minimum number of single-copy loci.

## Availability and Requirements

Project name: proTRAC

Project home pages:

https://sourceforge.net/projects/protrac/

http://www.uni-mainz.de/FB/Biologie/Anthropologie/472_ENG_HTML.php

Operating system(s): Platform independent (an executable file is available for Windows systems)

Programming language: Perl

Other requirements: Perl (no other requirements for executable file)

License: Academic Free License (AFL)

Any restrictions to use by non-academics: For commercial use please contact DR.

## Authors' contributions

DR is responsible for the development of the proTRAC software, performed the comparative analysis and drafted the manuscript. HZ participated in drafting the manuscript and made substantial contributions to the conception of the study. All authors read and approved the final manuscript.

## Supplementary Material

Additional file 1 **proTRAC output summary file that contains a list of all human piRNA clusters detected by proTRAC**. Click here for file

Additional file 2 **proTRAC output summary file that contains a list of all mouse piRNA clusters detected by proTRAC**. Click here for file

Additional file 3 **proTRAC output summary file that contains a list of all rat piRNA clusters detected by proTRAC**. Click here for file

Additional file 4 **proTRAC results folder containing a picture and a FASTA file for each detected human cluster**. After decompression, the folder can be opened as former session in proTRAC. Alternatively, each file can be opened separately with any standard text-editor or graphic-viewer respectively. Click here for file

Additional file 5 **proTRAC results folder containing a picture and a FASTA file for each detected mouse cluster**. After decompression, the folder can be opened as former session in proTRAC. Alternatively, each file can be opened separately with any standard text-editor or graphic-viewer respectively. Click here for file

Additional file 6 **proTRAC results folder containing a picture and a FASTA file for each detected rat cluster**. After decompression, the folder can be opened as former session in proTRAC. Alternatively, each file can be opened separately with any standard text-editor or graphic-viewer respectively. Click here for file

Additional file 7 **This document contains information on how the *Macaca mulatta *small RNA library was prepared and sequenced**. Click here for file

Additional file 8 **proTRAC results folder containing a picture and a FASTA file for each detected macaca cluster**. After decompression, the folder can be opened as former session in proTRAC. Alternatively, each file can be opened separately with any standard text-editor or graphic-viewer respectively. Click here for file

Additional file 9 **This folder contains the proTRAC software with all required files and a sample ELAND3 input file**. The Perl script (proTRAC.pl) contains the source code of the software that can be run on any platform. Executing Perl scripts requires the installation of a Perl interpreter which is part of a standard Perl distribution like the freely available Strawberry Perl (http://strawberryperl.com/). Perl is preinstalled on most Macintosh and Linux systems. The folder also contains an executable file (proTRAC.exe) which runs on Windows systems without any further requirements. Click here for file
