# Supplementary figures and images for: proTRAC - a software for probabilistic piRNA cluster detection, visualization and analysis (part 2 of 4)
Source: BMC Bioinformatics. 2012 Jan 10;13:5. doi: 10.1186/1471-2105-13-5 (PMC3293768; doi:10.1186/1471-2105-13-5)

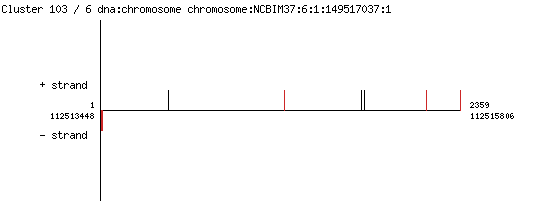

Supplement: Additional file 5 — proTRAC results folder containing a picture and a FASTA file for each detected mouse cluster. After decompression, the folder can be opened as former session in proTRAC. Alternatively, each file can be opened separately with any standard text-editor or graphic-viewer respectively. [file 1471-2105-13-5-S5.ZIP › proTRAC_results_mouse/bidirectional_clusters/Cluster_103.png]

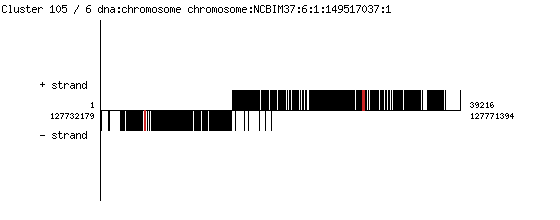

Supplement: Additional file 5 — proTRAC results folder containing a picture and a FASTA file for each detected mouse cluster. After decompression, the folder can be opened as former session in proTRAC. Alternatively, each file can be opened separately with any standard text-editor or graphic-viewer respectively. [file 1471-2105-13-5-S5.ZIP › proTRAC_results_mouse/bidirectional_clusters/Cluster_105.png]

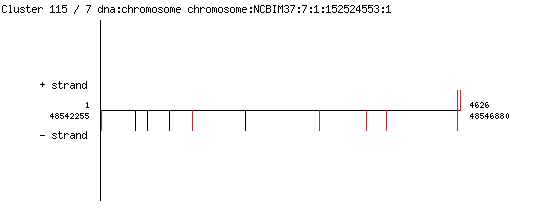

Supplement: Additional file 5 — proTRAC results folder containing a picture and a FASTA file for each detected mouse cluster. After decompression, the folder can be opened as former session in proTRAC. Alternatively, each file can be opened separately with any standard text-editor or graphic-viewer respectively. [file 1471-2105-13-5-S5.ZIP › proTRAC_results_mouse/bidirectional_clusters/Cluster_115.png]

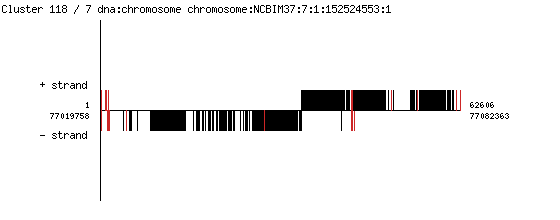

Supplement: Additional file 5 — proTRAC results folder containing a picture and a FASTA file for each detected mouse cluster. After decompression, the folder can be opened as former session in proTRAC. Alternatively, each file can be opened separately with any standard text-editor or graphic-viewer respectively. [file 1471-2105-13-5-S5.ZIP › proTRAC_results_mouse/bidirectional_clusters/Cluster_118.png]

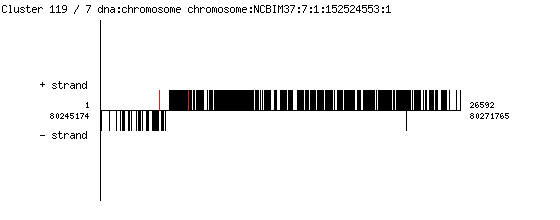

Supplement: Additional file 5 — proTRAC results folder containing a picture and a FASTA file for each detected mouse cluster. After decompression, the folder can be opened as former session in proTRAC. Alternatively, each file can be opened separately with any standard text-editor or graphic-viewer respectively. [file 1471-2105-13-5-S5.ZIP › proTRAC_results_mouse/bidirectional_clusters/Cluster_119.png]

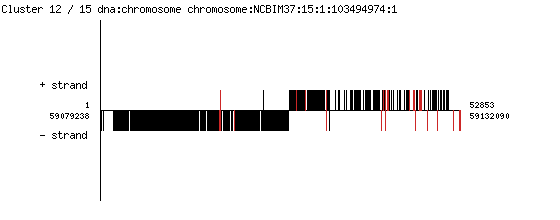

Supplement: Additional file 5 — proTRAC results folder containing a picture and a FASTA file for each detected mouse cluster. After decompression, the folder can be opened as former session in proTRAC. Alternatively, each file can be opened separately with any standard text-editor or graphic-viewer respectively. [file 1471-2105-13-5-S5.ZIP › proTRAC_results_mouse/bidirectional_clusters/Cluster_12.png]

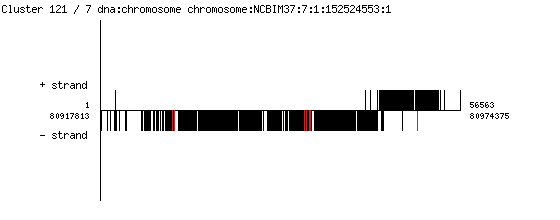

Supplement: Additional file 5 — proTRAC results folder containing a picture and a FASTA file for each detected mouse cluster. After decompression, the folder can be opened as former session in proTRAC. Alternatively, each file can be opened separately with any standard text-editor or graphic-viewer respectively. [file 1471-2105-13-5-S5.ZIP › proTRAC_results_mouse/bidirectional_clusters/Cluster_121.png]

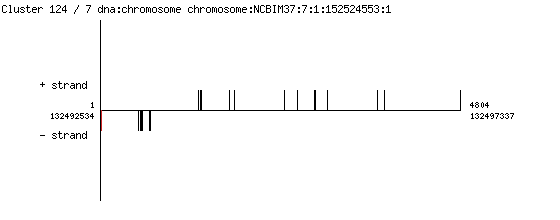

Supplement: Additional file 5 — proTRAC results folder containing a picture and a FASTA file for each detected mouse cluster. After decompression, the folder can be opened as former session in proTRAC. Alternatively, each file can be opened separately with any standard text-editor or graphic-viewer respectively. [file 1471-2105-13-5-S5.ZIP › proTRAC_results_mouse/bidirectional_clusters/Cluster_124.png]

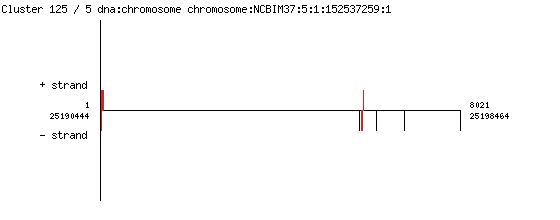

Supplement: Additional file 5 — proTRAC results folder containing a picture and a FASTA file for each detected mouse cluster. After decompression, the folder can be opened as former session in proTRAC. Alternatively, each file can be opened separately with any standard text-editor or graphic-viewer respectively. [file 1471-2105-13-5-S5.ZIP › proTRAC_results_mouse/bidirectional_clusters/Cluster_125.png]

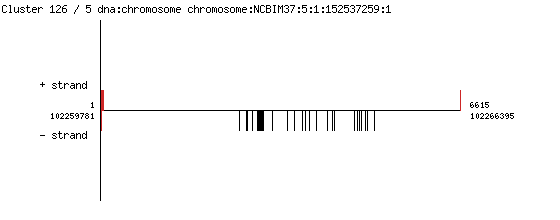

Supplement: Additional file 5 — proTRAC results folder containing a picture and a FASTA file for each detected mouse cluster. After decompression, the folder can be opened as former session in proTRAC. Alternatively, each file can be opened separately with any standard text-editor or graphic-viewer respectively. [file 1471-2105-13-5-S5.ZIP › proTRAC_results_mouse/bidirectional_clusters/Cluster_126.png]

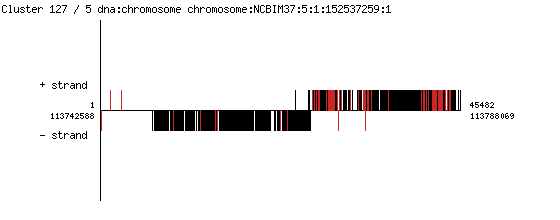

Supplement: Additional file 5 — proTRAC results folder containing a picture and a FASTA file for each detected mouse cluster. After decompression, the folder can be opened as former session in proTRAC. Alternatively, each file can be opened separately with any standard text-editor or graphic-viewer respectively. [file 1471-2105-13-5-S5.ZIP › proTRAC_results_mouse/bidirectional_clusters/Cluster_127.png]

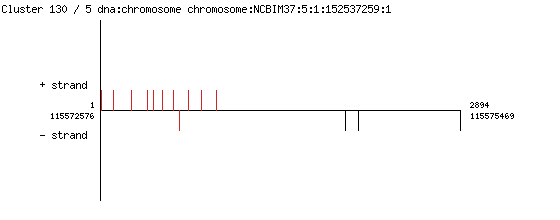

Supplement: Additional file 5 — proTRAC results folder containing a picture and a FASTA file for each detected mouse cluster. After decompression, the folder can be opened as former session in proTRAC. Alternatively, each file can be opened separately with any standard text-editor or graphic-viewer respectively. [file 1471-2105-13-5-S5.ZIP › proTRAC_results_mouse/bidirectional_clusters/Cluster_130.png]

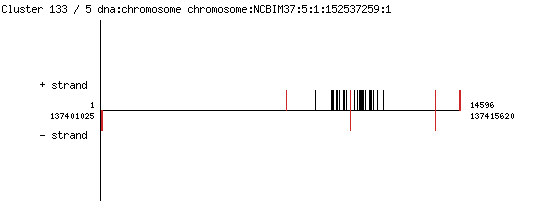

Supplement: Additional file 5 — proTRAC results folder containing a picture and a FASTA file for each detected mouse cluster. After decompression, the folder can be opened as former session in proTRAC. Alternatively, each file can be opened separately with any standard text-editor or graphic-viewer respectively. [file 1471-2105-13-5-S5.ZIP › proTRAC_results_mouse/bidirectional_clusters/Cluster_133.png]

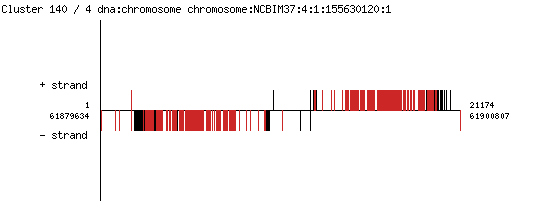

Supplement: Additional file 5 — proTRAC results folder containing a picture and a FASTA file for each detected mouse cluster. After decompression, the folder can be opened as former session in proTRAC. Alternatively, each file can be opened separately with any standard text-editor or graphic-viewer respectively. [file 1471-2105-13-5-S5.ZIP › proTRAC_results_mouse/bidirectional_clusters/Cluster_140.png]

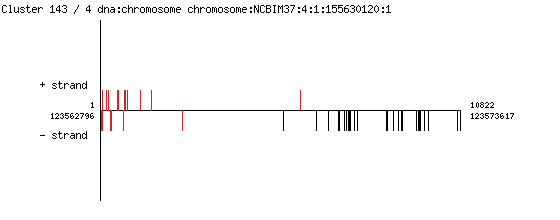

Supplement: Additional file 5 — proTRAC results folder containing a picture and a FASTA file for each detected mouse cluster. After decompression, the folder can be opened as former session in proTRAC. Alternatively, each file can be opened separately with any standard text-editor or graphic-viewer respectively. [file 1471-2105-13-5-S5.ZIP › proTRAC_results_mouse/bidirectional_clusters/Cluster_143.png]

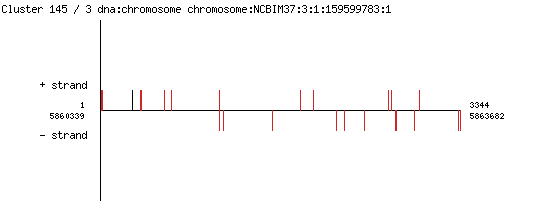

Supplement: Additional file 5 — proTRAC results folder containing a picture and a FASTA file for each detected mouse cluster. After decompression, the folder can be opened as former session in proTRAC. Alternatively, each file can be opened separately with any standard text-editor or graphic-viewer respectively. [file 1471-2105-13-5-S5.ZIP › proTRAC_results_mouse/bidirectional_clusters/Cluster_145.png]

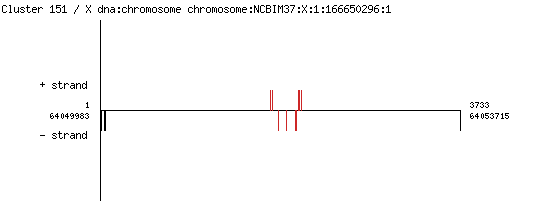

Supplement: Additional file 5 — proTRAC results folder containing a picture and a FASTA file for each detected mouse cluster. After decompression, the folder can be opened as former session in proTRAC. Alternatively, each file can be opened separately with any standard text-editor or graphic-viewer respectively. [file 1471-2105-13-5-S5.ZIP › proTRAC_results_mouse/bidirectional_clusters/Cluster_151.png]

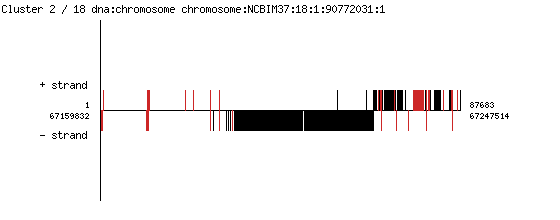

Supplement: Additional file 5 — proTRAC results folder containing a picture and a FASTA file for each detected mouse cluster. After decompression, the folder can be opened as former session in proTRAC. Alternatively, each file can be opened separately with any standard text-editor or graphic-viewer respectively. [file 1471-2105-13-5-S5.ZIP › proTRAC_results_mouse/bidirectional_clusters/Cluster_2.png]

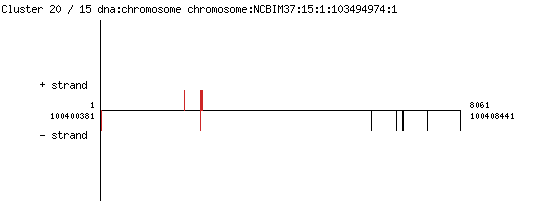

Supplement: Additional file 5 — proTRAC results folder containing a picture and a FASTA file for each detected mouse cluster. After decompression, the folder can be opened as former session in proTRAC. Alternatively, each file can be opened separately with any standard text-editor or graphic-viewer respectively. [file 1471-2105-13-5-S5.ZIP › proTRAC_results_mouse/bidirectional_clusters/Cluster_20.png]

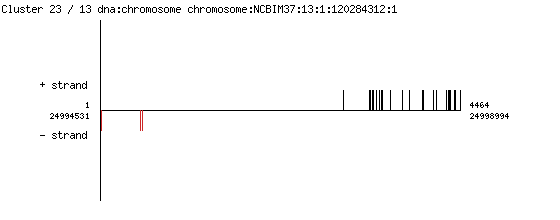

Supplement: Additional file 5 — proTRAC results folder containing a picture and a FASTA file for each detected mouse cluster. After decompression, the folder can be opened as former session in proTRAC. Alternatively, each file can be opened separately with any standard text-editor or graphic-viewer respectively. [file 1471-2105-13-5-S5.ZIP › proTRAC_results_mouse/bidirectional_clusters/Cluster_23.png]

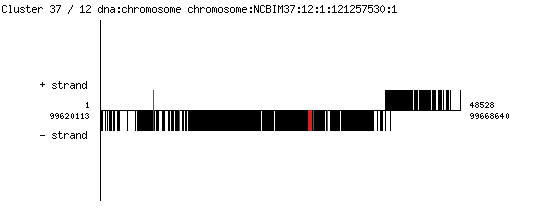

Supplement: Additional file 5 — proTRAC results folder containing a picture and a FASTA file for each detected mouse cluster. After decompression, the folder can be opened as former session in proTRAC. Alternatively, each file can be opened separately with any standard text-editor or graphic-viewer respectively. [file 1471-2105-13-5-S5.ZIP › proTRAC_results_mouse/bidirectional_clusters/Cluster_37.png]

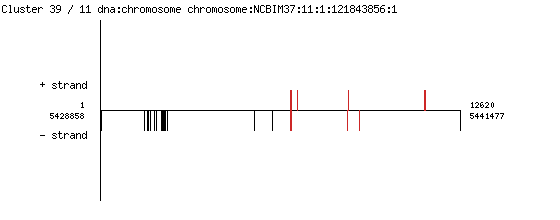

Supplement: Additional file 5 — proTRAC results folder containing a picture and a FASTA file for each detected mouse cluster. After decompression, the folder can be opened as former session in proTRAC. Alternatively, each file can be opened separately with any standard text-editor or graphic-viewer respectively. [file 1471-2105-13-5-S5.ZIP › proTRAC_results_mouse/bidirectional_clusters/Cluster_39.png]

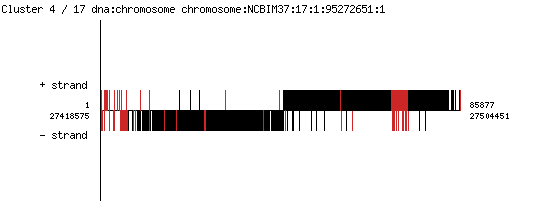

Supplement: Additional file 5 — proTRAC results folder containing a picture and a FASTA file for each detected mouse cluster. After decompression, the folder can be opened as former session in proTRAC. Alternatively, each file can be opened separately with any standard text-editor or graphic-viewer respectively. [file 1471-2105-13-5-S5.ZIP › proTRAC_results_mouse/bidirectional_clusters/Cluster_4.png]

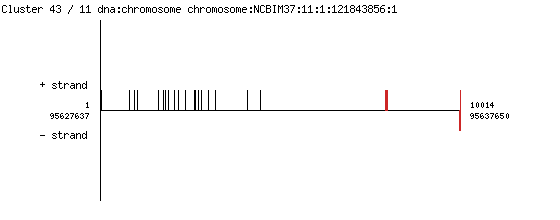

Supplement: Additional file 5 — proTRAC results folder containing a picture and a FASTA file for each detected mouse cluster. After decompression, the folder can be opened as former session in proTRAC. Alternatively, each file can be opened separately with any standard text-editor or graphic-viewer respectively. [file 1471-2105-13-5-S5.ZIP › proTRAC_results_mouse/bidirectional_clusters/Cluster_43.png]

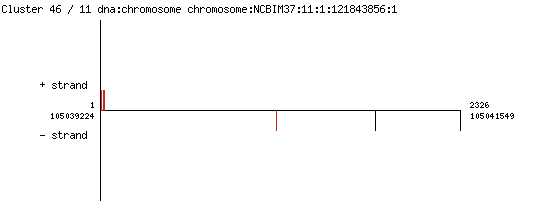

Supplement: Additional file 5 — proTRAC results folder containing a picture and a FASTA file for each detected mouse cluster. After decompression, the folder can be opened as former session in proTRAC. Alternatively, each file can be opened separately with any standard text-editor or graphic-viewer respectively. [file 1471-2105-13-5-S5.ZIP › proTRAC_results_mouse/bidirectional_clusters/Cluster_46.png]

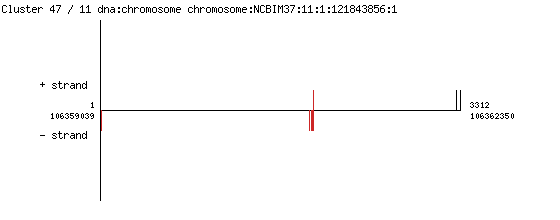

Supplement: Additional file 5 — proTRAC results folder containing a picture and a FASTA file for each detected mouse cluster. After decompression, the folder can be opened as former session in proTRAC. Alternatively, each file can be opened separately with any standard text-editor or graphic-viewer respectively. [file 1471-2105-13-5-S5.ZIP › proTRAC_results_mouse/bidirectional_clusters/Cluster_47.png]

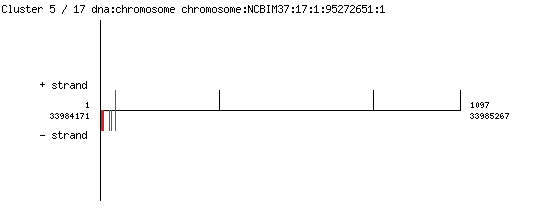

Supplement: Additional file 5 — proTRAC results folder containing a picture and a FASTA file for each detected mouse cluster. After decompression, the folder can be opened as former session in proTRAC. Alternatively, each file can be opened separately with any standard text-editor or graphic-viewer respectively. [file 1471-2105-13-5-S5.ZIP › proTRAC_results_mouse/bidirectional_clusters/Cluster_5.png]

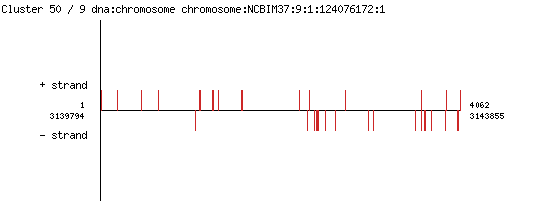

Supplement: Additional file 5 — proTRAC results folder containing a picture and a FASTA file for each detected mouse cluster. After decompression, the folder can be opened as former session in proTRAC. Alternatively, each file can be opened separately with any standard text-editor or graphic-viewer respectively. [file 1471-2105-13-5-S5.ZIP › proTRAC_results_mouse/bidirectional_clusters/Cluster_50.png]

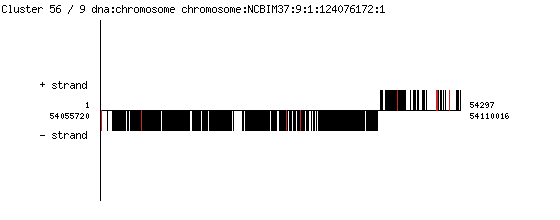

Supplement: Additional file 5 — proTRAC results folder containing a picture and a FASTA file for each detected mouse cluster. After decompression, the folder can be opened as former session in proTRAC. Alternatively, each file can be opened separately with any standard text-editor or graphic-viewer respectively. [file 1471-2105-13-5-S5.ZIP › proTRAC_results_mouse/bidirectional_clusters/Cluster_56.png]

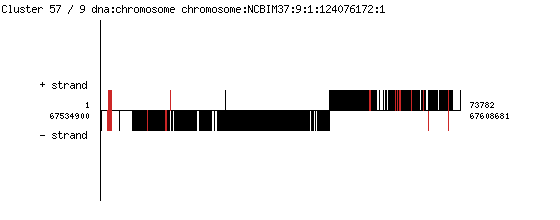

Supplement: Additional file 5 — proTRAC results folder containing a picture and a FASTA file for each detected mouse cluster. After decompression, the folder can be opened as former session in proTRAC. Alternatively, each file can be opened separately with any standard text-editor or graphic-viewer respectively. [file 1471-2105-13-5-S5.ZIP › proTRAC_results_mouse/bidirectional_clusters/Cluster_57.png]

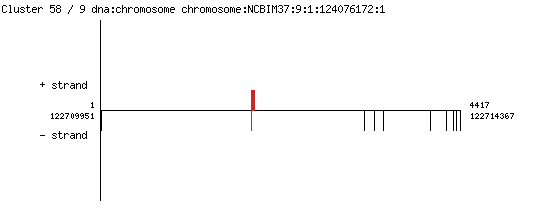

Supplement: Additional file 5 — proTRAC results folder containing a picture and a FASTA file for each detected mouse cluster. After decompression, the folder can be opened as former session in proTRAC. Alternatively, each file can be opened separately with any standard text-editor or graphic-viewer respectively. [file 1471-2105-13-5-S5.ZIP › proTRAC_results_mouse/bidirectional_clusters/Cluster_58.png]

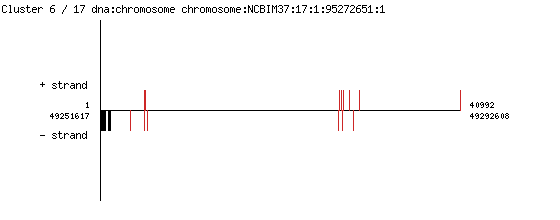

Supplement: Additional file 5 — proTRAC results folder containing a picture and a FASTA file for each detected mouse cluster. After decompression, the folder can be opened as former session in proTRAC. Alternatively, each file can be opened separately with any standard text-editor or graphic-viewer respectively. [file 1471-2105-13-5-S5.ZIP › proTRAC_results_mouse/bidirectional_clusters/Cluster_6.png]

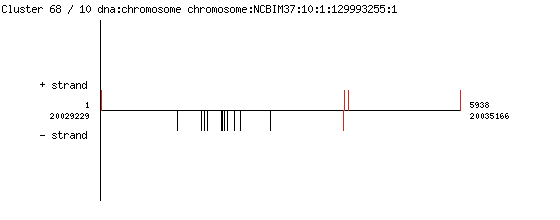

Supplement: Additional file 5 — proTRAC results folder containing a picture and a FASTA file for each detected mouse cluster. After decompression, the folder can be opened as former session in proTRAC. Alternatively, each file can be opened separately with any standard text-editor or graphic-viewer respectively. [file 1471-2105-13-5-S5.ZIP › proTRAC_results_mouse/bidirectional_clusters/Cluster_68.png]

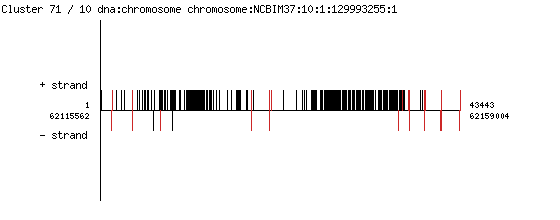

Supplement: Additional file 5 — proTRAC results folder containing a picture and a FASTA file for each detected mouse cluster. After decompression, the folder can be opened as former session in proTRAC. Alternatively, each file can be opened separately with any standard text-editor or graphic-viewer respectively. [file 1471-2105-13-5-S5.ZIP › proTRAC_results_mouse/bidirectional_clusters/Cluster_71.png]

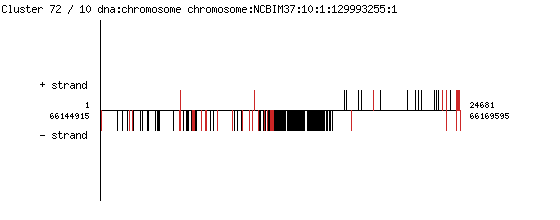

Supplement: Additional file 5 — proTRAC results folder containing a picture and a FASTA file for each detected mouse cluster. After decompression, the folder can be opened as former session in proTRAC. Alternatively, each file can be opened separately with any standard text-editor or graphic-viewer respectively. [file 1471-2105-13-5-S5.ZIP › proTRAC_results_mouse/bidirectional_clusters/Cluster_72.png]

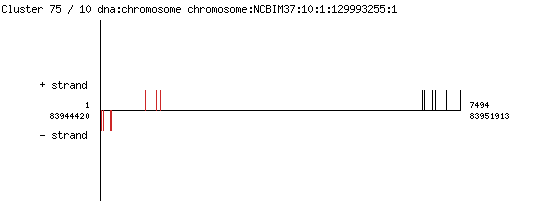

Supplement: Additional file 5 — proTRAC results folder containing a picture and a FASTA file for each detected mouse cluster. After decompression, the folder can be opened as former session in proTRAC. Alternatively, each file can be opened separately with any standard text-editor or graphic-viewer respectively. [file 1471-2105-13-5-S5.ZIP › proTRAC_results_mouse/bidirectional_clusters/Cluster_75.png]

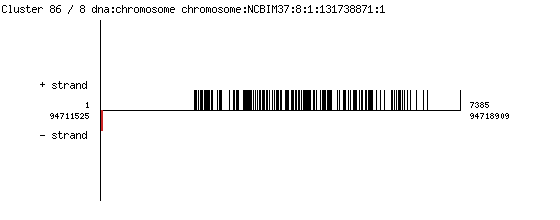

Supplement: Additional file 5 — proTRAC results folder containing a picture and a FASTA file for each detected mouse cluster. After decompression, the folder can be opened as former session in proTRAC. Alternatively, each file can be opened separately with any standard text-editor or graphic-viewer respectively. [file 1471-2105-13-5-S5.ZIP › proTRAC_results_mouse/bidirectional_clusters/Cluster_86.png]

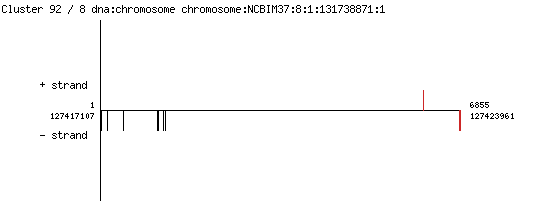

Supplement: Additional file 5 — proTRAC results folder containing a picture and a FASTA file for each detected mouse cluster. After decompression, the folder can be opened as former session in proTRAC. Alternatively, each file can be opened separately with any standard text-editor or graphic-viewer respectively. [file 1471-2105-13-5-S5.ZIP › proTRAC_results_mouse/bidirectional_clusters/Cluster_92.png]

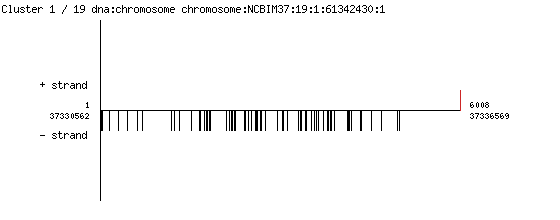

Supplement: Additional file 5 — proTRAC results folder containing a picture and a FASTA file for each detected mouse cluster. After decompression, the folder can be opened as former session in proTRAC. Alternatively, each file can be opened separately with any standard text-editor or graphic-viewer respectively. [file 1471-2105-13-5-S5.ZIP › proTRAC_results_mouse/monodirectional_clusters/Cluster_1.png]

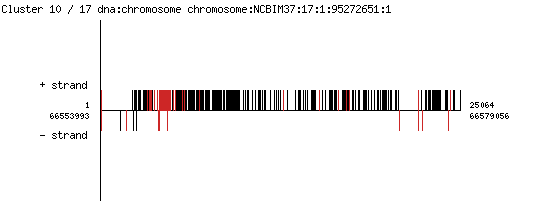

Supplement: Additional file 5 — proTRAC results folder containing a picture and a FASTA file for each detected mouse cluster. After decompression, the folder can be opened as former session in proTRAC. Alternatively, each file can be opened separately with any standard text-editor or graphic-viewer respectively. [file 1471-2105-13-5-S5.ZIP › proTRAC_results_mouse/monodirectional_clusters/Cluster_10.png]

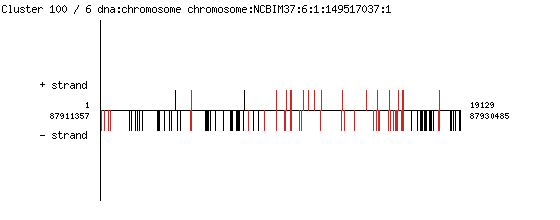

Supplement: Additional file 5 — proTRAC results folder containing a picture and a FASTA file for each detected mouse cluster. After decompression, the folder can be opened as former session in proTRAC. Alternatively, each file can be opened separately with any standard text-editor or graphic-viewer respectively. [file 1471-2105-13-5-S5.ZIP › proTRAC_results_mouse/monodirectional_clusters/Cluster_100.png]

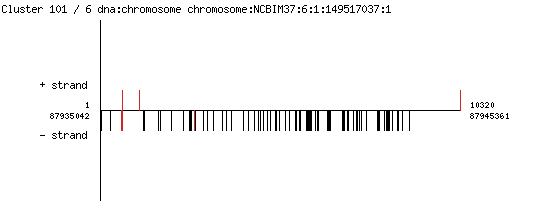

Supplement: Additional file 5 — proTRAC results folder containing a picture and a FASTA file for each detected mouse cluster. After decompression, the folder can be opened as former session in proTRAC. Alternatively, each file can be opened separately with any standard text-editor or graphic-viewer respectively. [file 1471-2105-13-5-S5.ZIP › proTRAC_results_mouse/monodirectional_clusters/Cluster_101.png]

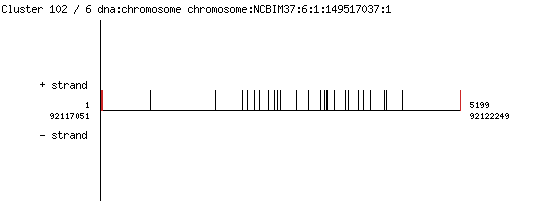

Supplement: Additional file 5 — proTRAC results folder containing a picture and a FASTA file for each detected mouse cluster. After decompression, the folder can be opened as former session in proTRAC. Alternatively, each file can be opened separately with any standard text-editor or graphic-viewer respectively. [file 1471-2105-13-5-S5.ZIP › proTRAC_results_mouse/monodirectional_clusters/Cluster_102.png]

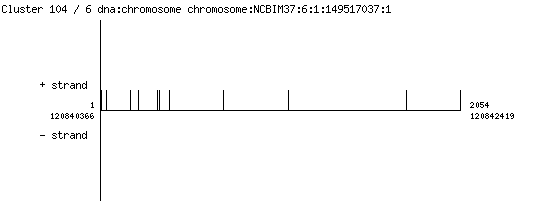

Supplement: Additional file 5 — proTRAC results folder containing a picture and a FASTA file for each detected mouse cluster. After decompression, the folder can be opened as former session in proTRAC. Alternatively, each file can be opened separately with any standard text-editor or graphic-viewer respectively. [file 1471-2105-13-5-S5.ZIP › proTRAC_results_mouse/monodirectional_clusters/Cluster_104.png]

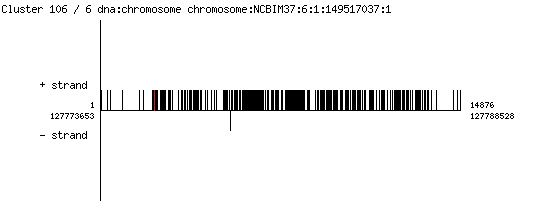

Supplement: Additional file 5 — proTRAC results folder containing a picture and a FASTA file for each detected mouse cluster. After decompression, the folder can be opened as former session in proTRAC. Alternatively, each file can be opened separately with any standard text-editor or graphic-viewer respectively. [file 1471-2105-13-5-S5.ZIP › proTRAC_results_mouse/monodirectional_clusters/Cluster_106.png]

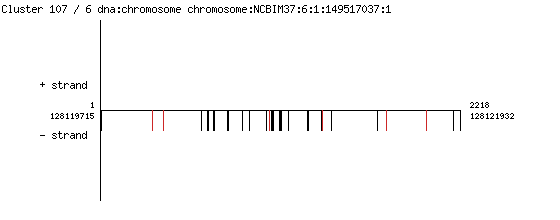

Supplement: Additional file 5 — proTRAC results folder containing a picture and a FASTA file for each detected mouse cluster. After decompression, the folder can be opened as former session in proTRAC. Alternatively, each file can be opened separately with any standard text-editor or graphic-viewer respectively. [file 1471-2105-13-5-S5.ZIP › proTRAC_results_mouse/monodirectional_clusters/Cluster_107.png]

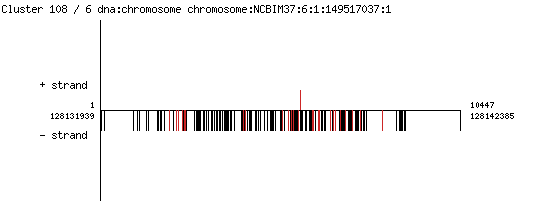

Supplement: Additional file 5 — proTRAC results folder containing a picture and a FASTA file for each detected mouse cluster. After decompression, the folder can be opened as former session in proTRAC. Alternatively, each file can be opened separately with any standard text-editor or graphic-viewer respectively. [file 1471-2105-13-5-S5.ZIP › proTRAC_results_mouse/monodirectional_clusters/Cluster_108.png]

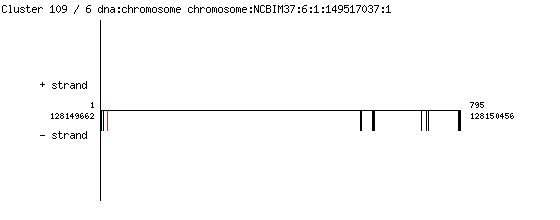

Supplement: Additional file 5 — proTRAC results folder containing a picture and a FASTA file for each detected mouse cluster. After decompression, the folder can be opened as former session in proTRAC. Alternatively, each file can be opened separately with any standard text-editor or graphic-viewer respectively. [file 1471-2105-13-5-S5.ZIP › proTRAC_results_mouse/monodirectional_clusters/Cluster_109.png]

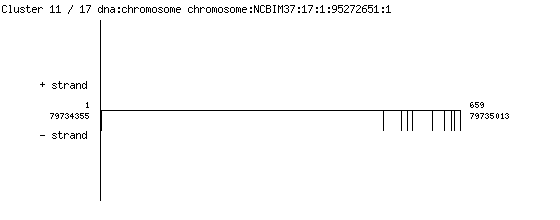

Supplement: Additional file 5 — proTRAC results folder containing a picture and a FASTA file for each detected mouse cluster. After decompression, the folder can be opened as former session in proTRAC. Alternatively, each file can be opened separately with any standard text-editor or graphic-viewer respectively. [file 1471-2105-13-5-S5.ZIP › proTRAC_results_mouse/monodirectional_clusters/Cluster_11.png]

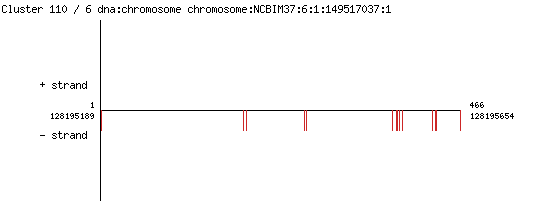

Supplement: Additional file 5 — proTRAC results folder containing a picture and a FASTA file for each detected mouse cluster. After decompression, the folder can be opened as former session in proTRAC. Alternatively, each file can be opened separately with any standard text-editor or graphic-viewer respectively. [file 1471-2105-13-5-S5.ZIP › proTRAC_results_mouse/monodirectional_clusters/Cluster_110.png]

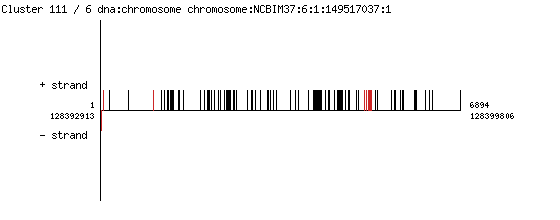

Supplement: Additional file 5 — proTRAC results folder containing a picture and a FASTA file for each detected mouse cluster. After decompression, the folder can be opened as former session in proTRAC. Alternatively, each file can be opened separately with any standard text-editor or graphic-viewer respectively. [file 1471-2105-13-5-S5.ZIP › proTRAC_results_mouse/monodirectional_clusters/Cluster_111.png]

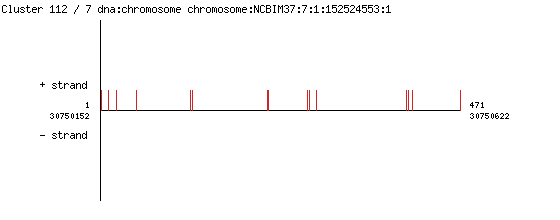

Supplement: Additional file 5 — proTRAC results folder containing a picture and a FASTA file for each detected mouse cluster. After decompression, the folder can be opened as former session in proTRAC. Alternatively, each file can be opened separately with any standard text-editor or graphic-viewer respectively. [file 1471-2105-13-5-S5.ZIP › proTRAC_results_mouse/monodirectional_clusters/Cluster_112.png]

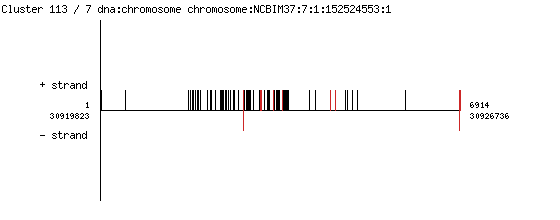

Supplement: Additional file 5 — proTRAC results folder containing a picture and a FASTA file for each detected mouse cluster. After decompression, the folder can be opened as former session in proTRAC. Alternatively, each file can be opened separately with any standard text-editor or graphic-viewer respectively. [file 1471-2105-13-5-S5.ZIP › proTRAC_results_mouse/monodirectional_clusters/Cluster_113.png]

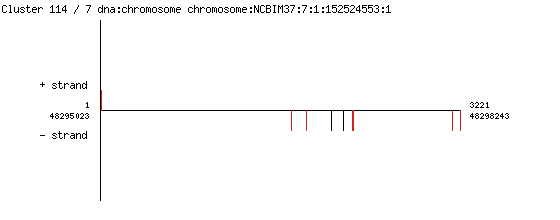

Supplement: Additional file 5 — proTRAC results folder containing a picture and a FASTA file for each detected mouse cluster. After decompression, the folder can be opened as former session in proTRAC. Alternatively, each file can be opened separately with any standard text-editor or graphic-viewer respectively. [file 1471-2105-13-5-S5.ZIP › proTRAC_results_mouse/monodirectional_clusters/Cluster_114.png]

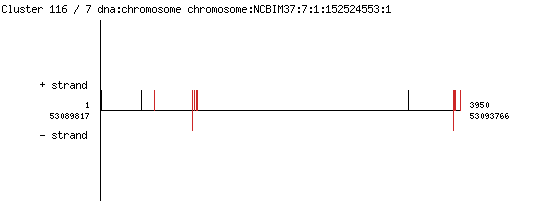

Supplement: Additional file 5 — proTRAC results folder containing a picture and a FASTA file for each detected mouse cluster. After decompression, the folder can be opened as former session in proTRAC. Alternatively, each file can be opened separately with any standard text-editor or graphic-viewer respectively. [file 1471-2105-13-5-S5.ZIP › proTRAC_results_mouse/monodirectional_clusters/Cluster_116.png]

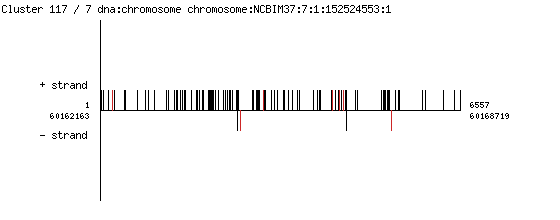

Supplement: Additional file 5 — proTRAC results folder containing a picture and a FASTA file for each detected mouse cluster. After decompression, the folder can be opened as former session in proTRAC. Alternatively, each file can be opened separately with any standard text-editor or graphic-viewer respectively. [file 1471-2105-13-5-S5.ZIP › proTRAC_results_mouse/monodirectional_clusters/Cluster_117.png]

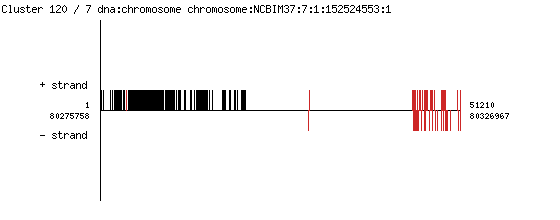

Supplement: Additional file 5 — proTRAC results folder containing a picture and a FASTA file for each detected mouse cluster. After decompression, the folder can be opened as former session in proTRAC. Alternatively, each file can be opened separately with any standard text-editor or graphic-viewer respectively. [file 1471-2105-13-5-S5.ZIP › proTRAC_results_mouse/monodirectional_clusters/Cluster_120.png]

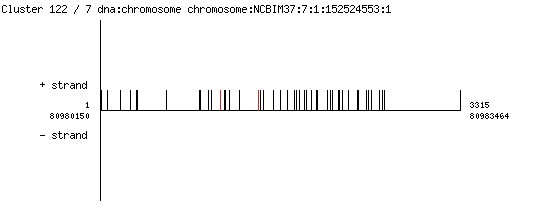

Supplement: Additional file 5 — proTRAC results folder containing a picture and a FASTA file for each detected mouse cluster. After decompression, the folder can be opened as former session in proTRAC. Alternatively, each file can be opened separately with any standard text-editor or graphic-viewer respectively. [file 1471-2105-13-5-S5.ZIP › proTRAC_results_mouse/monodirectional_clusters/Cluster_122.png]

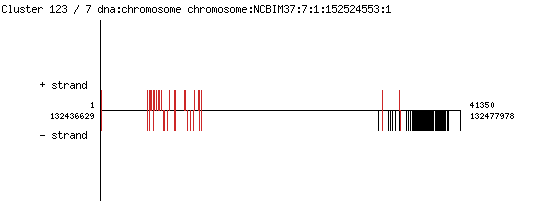

Supplement: Additional file 5 — proTRAC results folder containing a picture and a FASTA file for each detected mouse cluster. After decompression, the folder can be opened as former session in proTRAC. Alternatively, each file can be opened separately with any standard text-editor or graphic-viewer respectively. [file 1471-2105-13-5-S5.ZIP › proTRAC_results_mouse/monodirectional_clusters/Cluster_123.png]

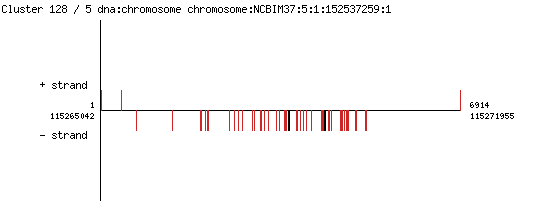

Supplement: Additional file 5 — proTRAC results folder containing a picture and a FASTA file for each detected mouse cluster. After decompression, the folder can be opened as former session in proTRAC. Alternatively, each file can be opened separately with any standard text-editor or graphic-viewer respectively. [file 1471-2105-13-5-S5.ZIP › proTRAC_results_mouse/monodirectional_clusters/Cluster_128.png]

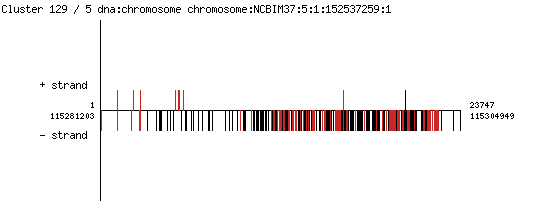

Supplement: Additional file 5 — proTRAC results folder containing a picture and a FASTA file for each detected mouse cluster. After decompression, the folder can be opened as former session in proTRAC. Alternatively, each file can be opened separately with any standard text-editor or graphic-viewer respectively. [file 1471-2105-13-5-S5.ZIP › proTRAC_results_mouse/monodirectional_clusters/Cluster_129.png]

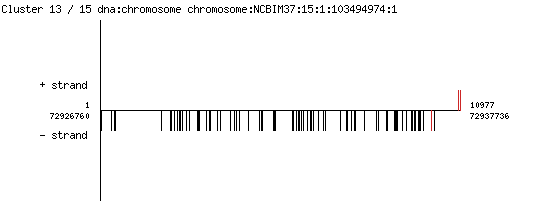

Supplement: Additional file 5 — proTRAC results folder containing a picture and a FASTA file for each detected mouse cluster. After decompression, the folder can be opened as former session in proTRAC. Alternatively, each file can be opened separately with any standard text-editor or graphic-viewer respectively. [file 1471-2105-13-5-S5.ZIP › proTRAC_results_mouse/monodirectional_clusters/Cluster_13.png]

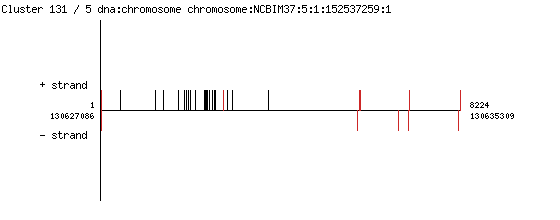

Supplement: Additional file 5 — proTRAC results folder containing a picture and a FASTA file for each detected mouse cluster. After decompression, the folder can be opened as former session in proTRAC. Alternatively, each file can be opened separately with any standard text-editor or graphic-viewer respectively. [file 1471-2105-13-5-S5.ZIP › proTRAC_results_mouse/monodirectional_clusters/Cluster_131.png]

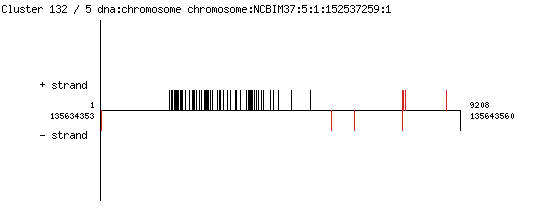

Supplement: Additional file 5 — proTRAC results folder containing a picture and a FASTA file for each detected mouse cluster. After decompression, the folder can be opened as former session in proTRAC. Alternatively, each file can be opened separately with any standard text-editor or graphic-viewer respectively. [file 1471-2105-13-5-S5.ZIP › proTRAC_results_mouse/monodirectional_clusters/Cluster_132.png]

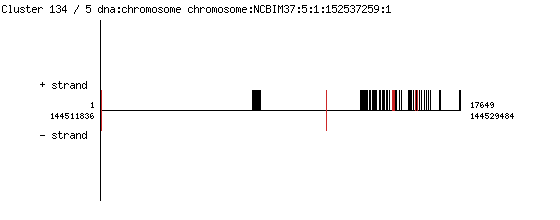

Supplement: Additional file 5 — proTRAC results folder containing a picture and a FASTA file for each detected mouse cluster. After decompression, the folder can be opened as former session in proTRAC. Alternatively, each file can be opened separately with any standard text-editor or graphic-viewer respectively. [file 1471-2105-13-5-S5.ZIP › proTRAC_results_mouse/monodirectional_clusters/Cluster_134.png]

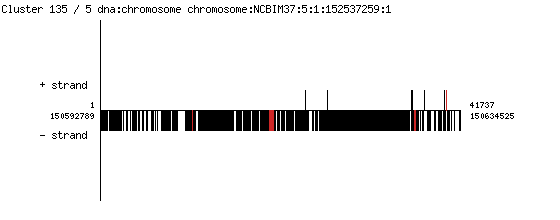

Supplement: Additional file 5 — proTRAC results folder containing a picture and a FASTA file for each detected mouse cluster. After decompression, the folder can be opened as former session in proTRAC. Alternatively, each file can be opened separately with any standard text-editor or graphic-viewer respectively. [file 1471-2105-13-5-S5.ZIP › proTRAC_results_mouse/monodirectional_clusters/Cluster_135.png]

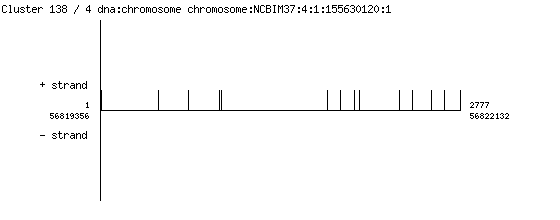

Supplement: Additional file 5 — proTRAC results folder containing a picture and a FASTA file for each detected mouse cluster. After decompression, the folder can be opened as former session in proTRAC. Alternatively, each file can be opened separately with any standard text-editor or graphic-viewer respectively. [file 1471-2105-13-5-S5.ZIP › proTRAC_results_mouse/monodirectional_clusters/Cluster_138.png]

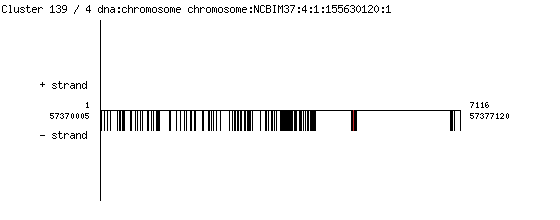

Supplement: Additional file 5 — proTRAC results folder containing a picture and a FASTA file for each detected mouse cluster. After decompression, the folder can be opened as former session in proTRAC. Alternatively, each file can be opened separately with any standard text-editor or graphic-viewer respectively. [file 1471-2105-13-5-S5.ZIP › proTRAC_results_mouse/monodirectional_clusters/Cluster_139.png]

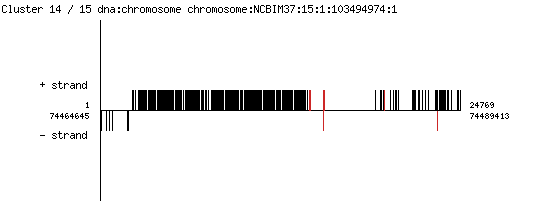

Supplement: Additional file 5 — proTRAC results folder containing a picture and a FASTA file for each detected mouse cluster. After decompression, the folder can be opened as former session in proTRAC. Alternatively, each file can be opened separately with any standard text-editor or graphic-viewer respectively. [file 1471-2105-13-5-S5.ZIP › proTRAC_results_mouse/monodirectional_clusters/Cluster_14.png]

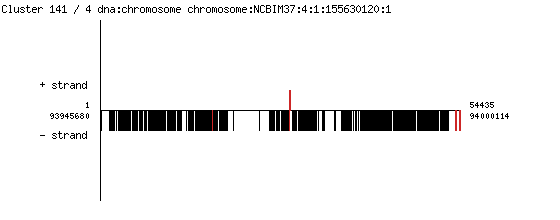

Supplement: Additional file 5 — proTRAC results folder containing a picture and a FASTA file for each detected mouse cluster. After decompression, the folder can be opened as former session in proTRAC. Alternatively, each file can be opened separately with any standard text-editor or graphic-viewer respectively. [file 1471-2105-13-5-S5.ZIP › proTRAC_results_mouse/monodirectional_clusters/Cluster_141.png]

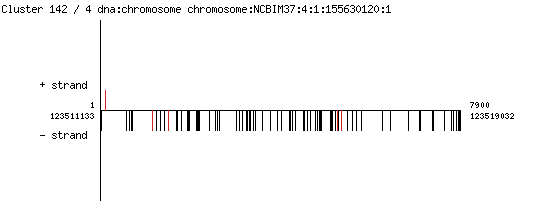

Supplement: Additional file 5 — proTRAC results folder containing a picture and a FASTA file for each detected mouse cluster. After decompression, the folder can be opened as former session in proTRAC. Alternatively, each file can be opened separately with any standard text-editor or graphic-viewer respectively. [file 1471-2105-13-5-S5.ZIP › proTRAC_results_mouse/monodirectional_clusters/Cluster_142.png]

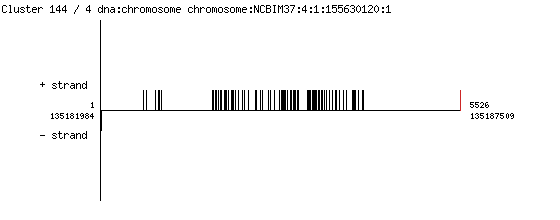

Supplement: Additional file 5 — proTRAC results folder containing a picture and a FASTA file for each detected mouse cluster. After decompression, the folder can be opened as former session in proTRAC. Alternatively, each file can be opened separately with any standard text-editor or graphic-viewer respectively. [file 1471-2105-13-5-S5.ZIP › proTRAC_results_mouse/monodirectional_clusters/Cluster_144.png]

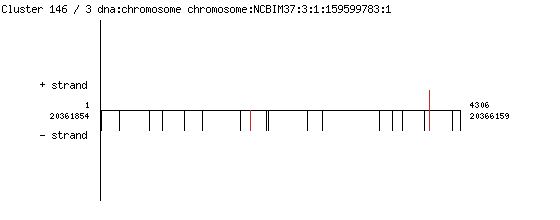

Supplement: Additional file 5 — proTRAC results folder containing a picture and a FASTA file for each detected mouse cluster. After decompression, the folder can be opened as former session in proTRAC. Alternatively, each file can be opened separately with any standard text-editor or graphic-viewer respectively. [file 1471-2105-13-5-S5.ZIP › proTRAC_results_mouse/monodirectional_clusters/Cluster_146.png]

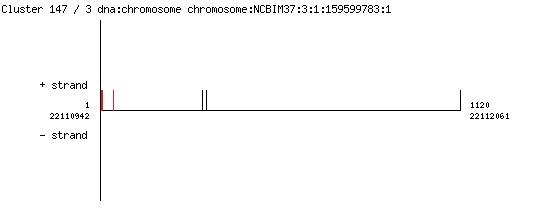

Supplement: Additional file 5 — proTRAC results folder containing a picture and a FASTA file for each detected mouse cluster. After decompression, the folder can be opened as former session in proTRAC. Alternatively, each file can be opened separately with any standard text-editor or graphic-viewer respectively. [file 1471-2105-13-5-S5.ZIP › proTRAC_results_mouse/monodirectional_clusters/Cluster_147.png]

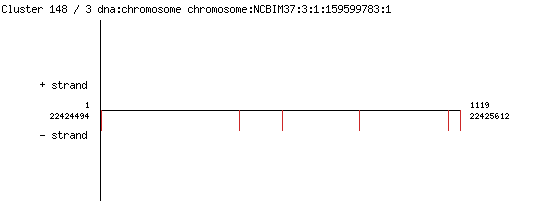

Supplement: Additional file 5 — proTRAC results folder containing a picture and a FASTA file for each detected mouse cluster. After decompression, the folder can be opened as former session in proTRAC. Alternatively, each file can be opened separately with any standard text-editor or graphic-viewer respectively. [file 1471-2105-13-5-S5.ZIP › proTRAC_results_mouse/monodirectional_clusters/Cluster_148.png]

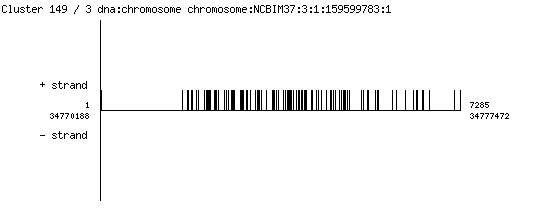

Supplement: Additional file 5 — proTRAC results folder containing a picture and a FASTA file for each detected mouse cluster. After decompression, the folder can be opened as former session in proTRAC. Alternatively, each file can be opened separately with any standard text-editor or graphic-viewer respectively. [file 1471-2105-13-5-S5.ZIP › proTRAC_results_mouse/monodirectional_clusters/Cluster_149.png]

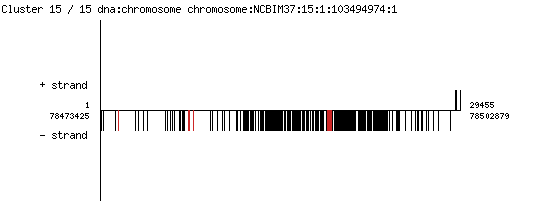

Supplement: Additional file 5 — proTRAC results folder containing a picture and a FASTA file for each detected mouse cluster. After decompression, the folder can be opened as former session in proTRAC. Alternatively, each file can be opened separately with any standard text-editor or graphic-viewer respectively. [file 1471-2105-13-5-S5.ZIP › proTRAC_results_mouse/monodirectional_clusters/Cluster_15.png]

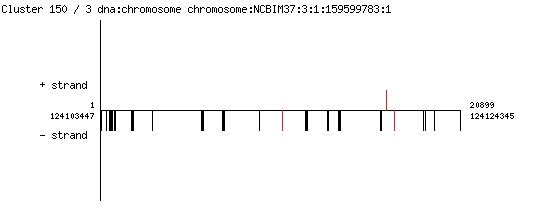

Supplement: Additional file 5 — proTRAC results folder containing a picture and a FASTA file for each detected mouse cluster. After decompression, the folder can be opened as former session in proTRAC. Alternatively, each file can be opened separately with any standard text-editor or graphic-viewer respectively. [file 1471-2105-13-5-S5.ZIP › proTRAC_results_mouse/monodirectional_clusters/Cluster_150.png]

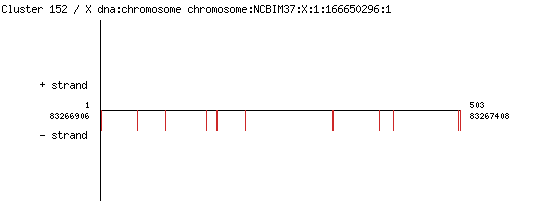

Supplement: Additional file 5 — proTRAC results folder containing a picture and a FASTA file for each detected mouse cluster. After decompression, the folder can be opened as former session in proTRAC. Alternatively, each file can be opened separately with any standard text-editor or graphic-viewer respectively. [file 1471-2105-13-5-S5.ZIP › proTRAC_results_mouse/monodirectional_clusters/Cluster_152.png]

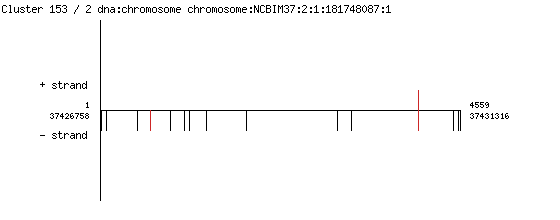

Supplement: Additional file 5 — proTRAC results folder containing a picture and a FASTA file for each detected mouse cluster. After decompression, the folder can be opened as former session in proTRAC. Alternatively, each file can be opened separately with any standard text-editor or graphic-viewer respectively. [file 1471-2105-13-5-S5.ZIP › proTRAC_results_mouse/monodirectional_clusters/Cluster_153.png]

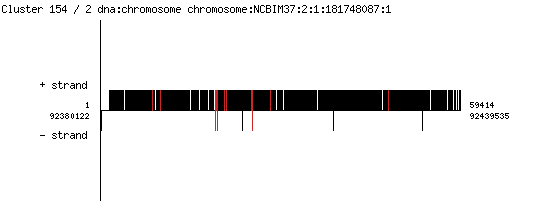

Supplement: Additional file 5 — proTRAC results folder containing a picture and a FASTA file for each detected mouse cluster. After decompression, the folder can be opened as former session in proTRAC. Alternatively, each file can be opened separately with any standard text-editor or graphic-viewer respectively. [file 1471-2105-13-5-S5.ZIP › proTRAC_results_mouse/monodirectional_clusters/Cluster_154.png]

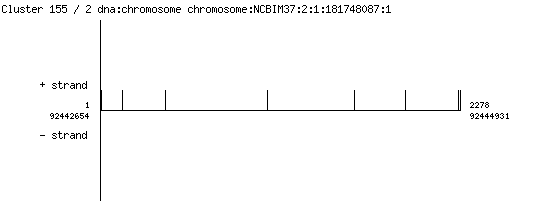

Supplement: Additional file 5 — proTRAC results folder containing a picture and a FASTA file for each detected mouse cluster. After decompression, the folder can be opened as former session in proTRAC. Alternatively, each file can be opened separately with any standard text-editor or graphic-viewer respectively. [file 1471-2105-13-5-S5.ZIP › proTRAC_results_mouse/monodirectional_clusters/Cluster_155.png]

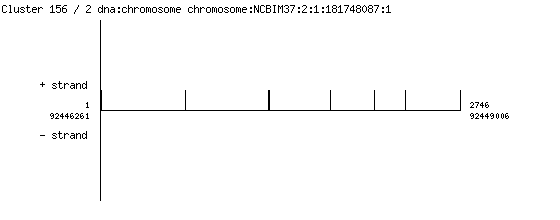

Supplement: Additional file 5 — proTRAC results folder containing a picture and a FASTA file for each detected mouse cluster. After decompression, the folder can be opened as former session in proTRAC. Alternatively, each file can be opened separately with any standard text-editor or graphic-viewer respectively. [file 1471-2105-13-5-S5.ZIP › proTRAC_results_mouse/monodirectional_clusters/Cluster_156.png]

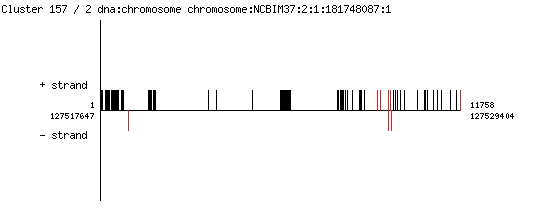

Supplement: Additional file 5 — proTRAC results folder containing a picture and a FASTA file for each detected mouse cluster. After decompression, the folder can be opened as former session in proTRAC. Alternatively, each file can be opened separately with any standard text-editor or graphic-viewer respectively. [file 1471-2105-13-5-S5.ZIP › proTRAC_results_mouse/monodirectional_clusters/Cluster_157.png]

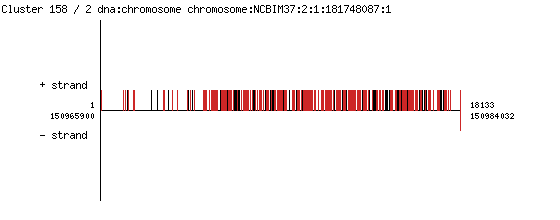

Supplement: Additional file 5 — proTRAC results folder containing a picture and a FASTA file for each detected mouse cluster. After decompression, the folder can be opened as former session in proTRAC. Alternatively, each file can be opened separately with any standard text-editor or graphic-viewer respectively. [file 1471-2105-13-5-S5.ZIP › proTRAC_results_mouse/monodirectional_clusters/Cluster_158.png]

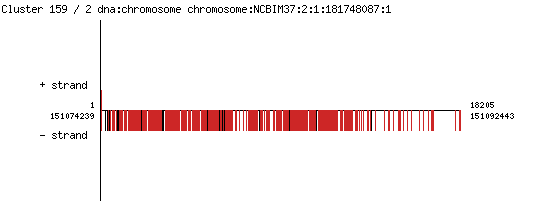

Supplement: Additional file 5 — proTRAC results folder containing a picture and a FASTA file for each detected mouse cluster. After decompression, the folder can be opened as former session in proTRAC. Alternatively, each file can be opened separately with any standard text-editor or graphic-viewer respectively. [file 1471-2105-13-5-S5.ZIP › proTRAC_results_mouse/monodirectional_clusters/Cluster_159.png]

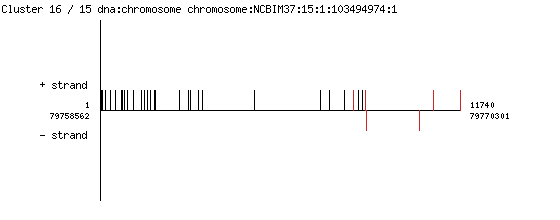

Supplement: Additional file 5 — proTRAC results folder containing a picture and a FASTA file for each detected mouse cluster. After decompression, the folder can be opened as former session in proTRAC. Alternatively, each file can be opened separately with any standard text-editor or graphic-viewer respectively. [file 1471-2105-13-5-S5.ZIP › proTRAC_results_mouse/monodirectional_clusters/Cluster_16.png]

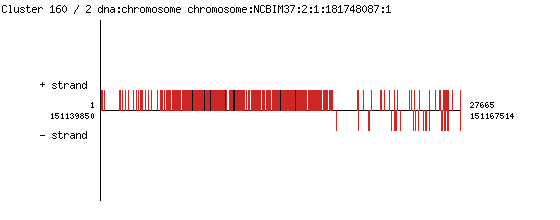

Supplement: Additional file 5 — proTRAC results folder containing a picture and a FASTA file for each detected mouse cluster. After decompression, the folder can be opened as former session in proTRAC. Alternatively, each file can be opened separately with any standard text-editor or graphic-viewer respectively. [file 1471-2105-13-5-S5.ZIP › proTRAC_results_mouse/monodirectional_clusters/Cluster_160.png]

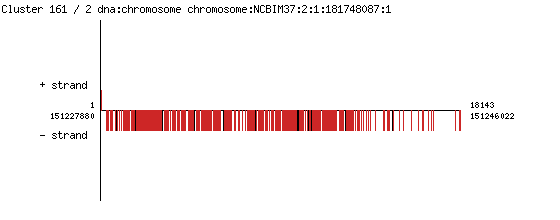

Supplement: Additional file 5 — proTRAC results folder containing a picture and a FASTA file for each detected mouse cluster. After decompression, the folder can be opened as former session in proTRAC. Alternatively, each file can be opened separately with any standard text-editor or graphic-viewer respectively. [file 1471-2105-13-5-S5.ZIP › proTRAC_results_mouse/monodirectional_clusters/Cluster_161.png]

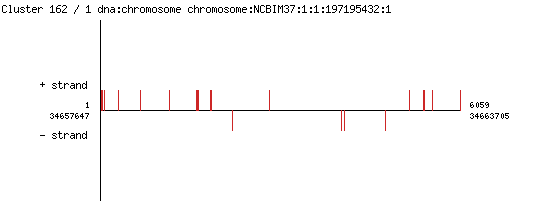

Supplement: Additional file 5 — proTRAC results folder containing a picture and a FASTA file for each detected mouse cluster. After decompression, the folder can be opened as former session in proTRAC. Alternatively, each file can be opened separately with any standard text-editor or graphic-viewer respectively. [file 1471-2105-13-5-S5.ZIP › proTRAC_results_mouse/monodirectional_clusters/Cluster_162.png]

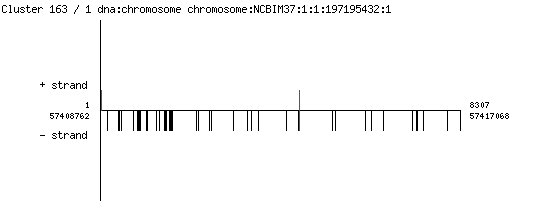

Supplement: Additional file 5 — proTRAC results folder containing a picture and a FASTA file for each detected mouse cluster. After decompression, the folder can be opened as former session in proTRAC. Alternatively, each file can be opened separately with any standard text-editor or graphic-viewer respectively. [file 1471-2105-13-5-S5.ZIP › proTRAC_results_mouse/monodirectional_clusters/Cluster_163.png]

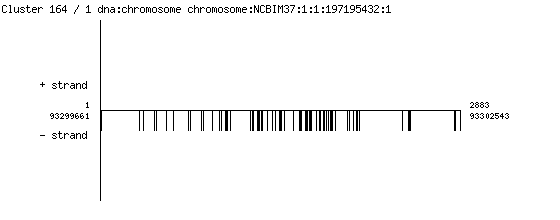

Supplement: Additional file 5 — proTRAC results folder containing a picture and a FASTA file for each detected mouse cluster. After decompression, the folder can be opened as former session in proTRAC. Alternatively, each file can be opened separately with any standard text-editor or graphic-viewer respectively. [file 1471-2105-13-5-S5.ZIP › proTRAC_results_mouse/monodirectional_clusters/Cluster_164.png]

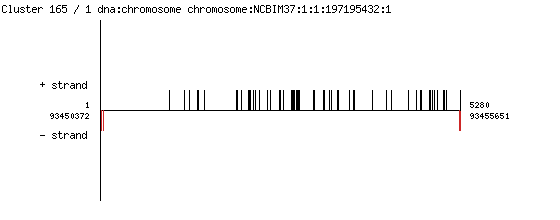

Supplement: Additional file 5 — proTRAC results folder containing a picture and a FASTA file for each detected mouse cluster. After decompression, the folder can be opened as former session in proTRAC. Alternatively, each file can be opened separately with any standard text-editor or graphic-viewer respectively. [file 1471-2105-13-5-S5.ZIP › proTRAC_results_mouse/monodirectional_clusters/Cluster_165.png]

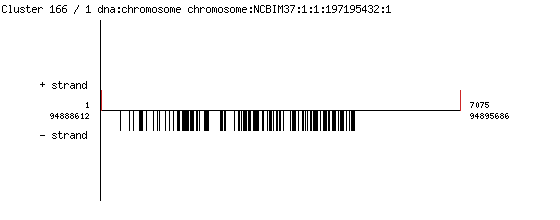

Supplement: Additional file 5 — proTRAC results folder containing a picture and a FASTA file for each detected mouse cluster. After decompression, the folder can be opened as former session in proTRAC. Alternatively, each file can be opened separately with any standard text-editor or graphic-viewer respectively. [file 1471-2105-13-5-S5.ZIP › proTRAC_results_mouse/monodirectional_clusters/Cluster_166.png]

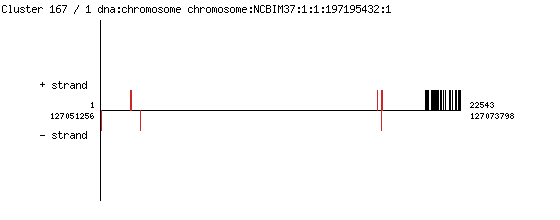

Supplement: Additional file 5 — proTRAC results folder containing a picture and a FASTA file for each detected mouse cluster. After decompression, the folder can be opened as former session in proTRAC. Alternatively, each file can be opened separately with any standard text-editor or graphic-viewer respectively. [file 1471-2105-13-5-S5.ZIP › proTRAC_results_mouse/monodirectional_clusters/Cluster_167.png]

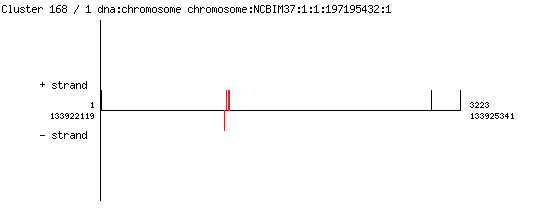

Supplement: Additional file 5 — proTRAC results folder containing a picture and a FASTA file for each detected mouse cluster. After decompression, the folder can be opened as former session in proTRAC. Alternatively, each file can be opened separately with any standard text-editor or graphic-viewer respectively. [file 1471-2105-13-5-S5.ZIP › proTRAC_results_mouse/monodirectional_clusters/Cluster_168.png]

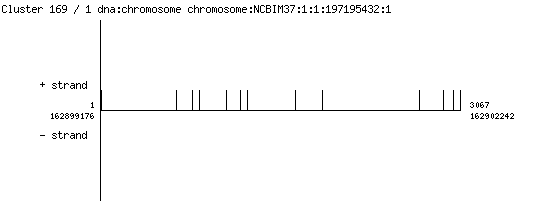

Supplement: Additional file 5 — proTRAC results folder containing a picture and a FASTA file for each detected mouse cluster. After decompression, the folder can be opened as former session in proTRAC. Alternatively, each file can be opened separately with any standard text-editor or graphic-viewer respectively. [file 1471-2105-13-5-S5.ZIP › proTRAC_results_mouse/monodirectional_clusters/Cluster_169.png]

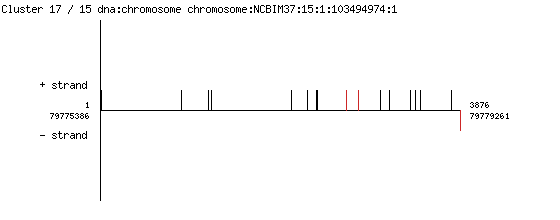

Supplement: Additional file 5 — proTRAC results folder containing a picture and a FASTA file for each detected mouse cluster. After decompression, the folder can be opened as former session in proTRAC. Alternatively, each file can be opened separately with any standard text-editor or graphic-viewer respectively. [file 1471-2105-13-5-S5.ZIP › proTRAC_results_mouse/monodirectional_clusters/Cluster_17.png]

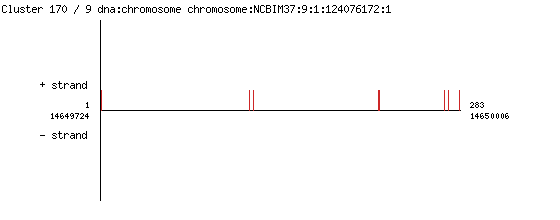

Supplement: Additional file 5 — proTRAC results folder containing a picture and a FASTA file for each detected mouse cluster. After decompression, the folder can be opened as former session in proTRAC. Alternatively, each file can be opened separately with any standard text-editor or graphic-viewer respectively. [file 1471-2105-13-5-S5.ZIP › proTRAC_results_mouse/monodirectional_clusters/Cluster_170.png]

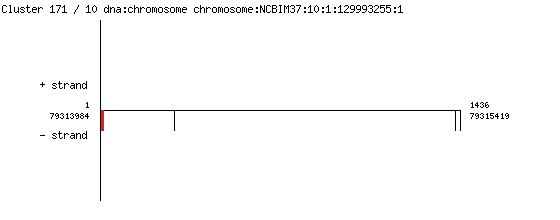

Supplement: Additional file 5 — proTRAC results folder containing a picture and a FASTA file for each detected mouse cluster. After decompression, the folder can be opened as former session in proTRAC. Alternatively, each file can be opened separately with any standard text-editor or graphic-viewer respectively. [file 1471-2105-13-5-S5.ZIP › proTRAC_results_mouse/monodirectional_clusters/Cluster_171.png]
