# Supplementary material for: proTRAC - a software for probabilistic piRNA cluster detection, visualization and analysis
Source: BMC Bioinformatics. 2012 Jan 10;13:5. doi: 10.1186/1471-2105-13-5 (PMC3293768; doi:10.1186/1471-2105-13-5)
Supplement: Additional file 7 — This document contains information on how the Macaca mulatta small RNA library was prepared and sequenced. [file 1471-2105-13-5-S7.DOC]

***Macaca mulatta* small RNA library preparation (from total testes RNA)**

Total RNA was purified from testes of *Macaca mulatta* with the TRIZOL (Invitrogen) method. Total RNA was loaded onto a 12% denaturing polyacrylamide gel and the 31-mer internal control RNA piSPIKE^TM^ (IDT®) was used to determine the piRNA containing fraction which was excised from the gel. RNA was eluted and purified using Ultrafree MC and Microcon-10 centrifugal filter devices (Millipore^TM^). Purified RNA was 3’-polyadenylated using A-Plus™ Poly(A) Polymerase Tailing Kit (Epicentre® Biotechnologies) and 5’-ligated with the RNA-linker rGrArCrUrGrGrArGrCrArCrGrArGrGrArCrArCrUrGrArArUrGrGrArCrUrGrArArGrGrArGrUrArGrArArA using T4 RNA Ligase. Reverse transcription (RT) was carried out with Superscript II Reverse Transcriptase (Invitrogen) and RT primer CGAATTCTAGAGCTCGAGGCAGGCGACATG(T)_25_NV. Finally, the reverse transcribed RNA was PCR-amplified using the Taq PCR Core Kit (Qiagen) with the primers GACTGGAGCACGAGGACACTGA and CGAATTCTAGAGCTCGAGGCAGG.

The obtained library was deep sequenced by Seq-IT GmbH & Co. KG (Kaiserslautern, Germany) using a 454 Genome Sequencer FLX system.
