# Supplementary figures and images for: proTRAC - a software for probabilistic piRNA cluster detection, visualization and analysis (part 4 of 4)
Source: BMC Bioinformatics. 2012 Jan 10;13:5. doi: 10.1186/1471-2105-13-5 (PMC3293768; doi:10.1186/1471-2105-13-5)

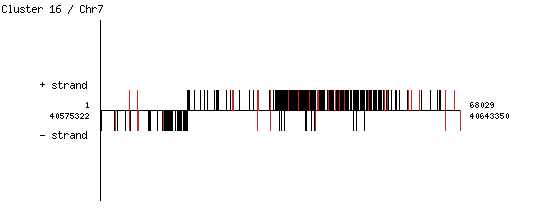

Supplement: Additional file 8 — proTRAC results folder containing a picture and a FASTA file for each detected macaca cluster. After decompression, the folder can be opened as former session in proTRAC. Alternatively, each file can be opened separately with any standard text-editor or graphic-viewer respectively. [file 1471-2105-13-5-S8.ZIP › proTRAC_results_macaca/bidirectional_clusters/Cluster_16.png]

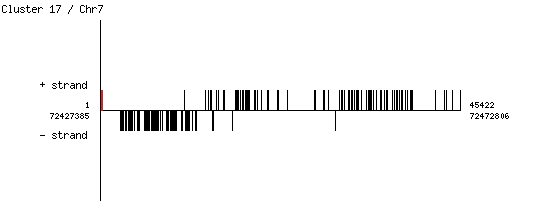

Supplement: Additional file 8 — proTRAC results folder containing a picture and a FASTA file for each detected macaca cluster. After decompression, the folder can be opened as former session in proTRAC. Alternatively, each file can be opened separately with any standard text-editor or graphic-viewer respectively. [file 1471-2105-13-5-S8.ZIP › proTRAC_results_macaca/bidirectional_clusters/Cluster_17.png]

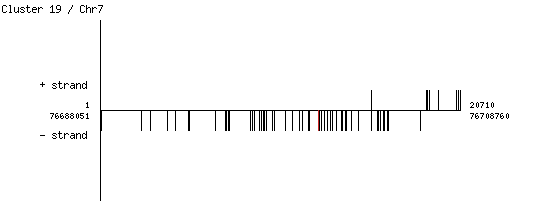

Supplement: Additional file 8 — proTRAC results folder containing a picture and a FASTA file for each detected macaca cluster. After decompression, the folder can be opened as former session in proTRAC. Alternatively, each file can be opened separately with any standard text-editor or graphic-viewer respectively. [file 1471-2105-13-5-S8.ZIP › proTRAC_results_macaca/bidirectional_clusters/Cluster_19.png]

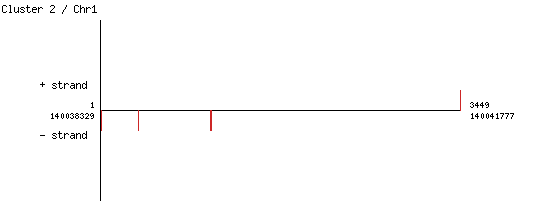

Supplement: Additional file 8 — proTRAC results folder containing a picture and a FASTA file for each detected macaca cluster. After decompression, the folder can be opened as former session in proTRAC. Alternatively, each file can be opened separately with any standard text-editor or graphic-viewer respectively. [file 1471-2105-13-5-S8.ZIP › proTRAC_results_macaca/bidirectional_clusters/Cluster_2.png]

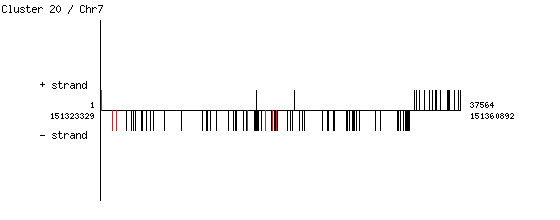

Supplement: Additional file 8 — proTRAC results folder containing a picture and a FASTA file for each detected macaca cluster. After decompression, the folder can be opened as former session in proTRAC. Alternatively, each file can be opened separately with any standard text-editor or graphic-viewer respectively. [file 1471-2105-13-5-S8.ZIP › proTRAC_results_macaca/bidirectional_clusters/Cluster_20.png]

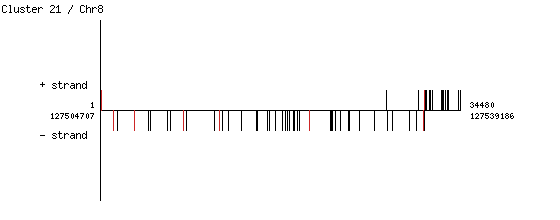

Supplement: Additional file 8 — proTRAC results folder containing a picture and a FASTA file for each detected macaca cluster. After decompression, the folder can be opened as former session in proTRAC. Alternatively, each file can be opened separately with any standard text-editor or graphic-viewer respectively. [file 1471-2105-13-5-S8.ZIP › proTRAC_results_macaca/bidirectional_clusters/Cluster_21.png]

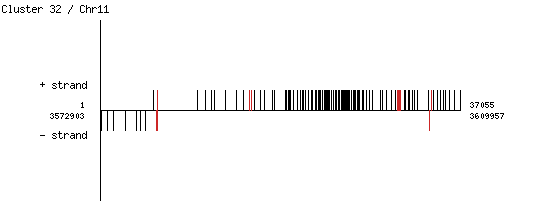

Supplement: Additional file 8 — proTRAC results folder containing a picture and a FASTA file for each detected macaca cluster. After decompression, the folder can be opened as former session in proTRAC. Alternatively, each file can be opened separately with any standard text-editor or graphic-viewer respectively. [file 1471-2105-13-5-S8.ZIP › proTRAC_results_macaca/bidirectional_clusters/Cluster_32.png]

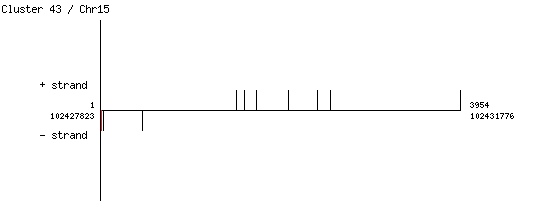

Supplement: Additional file 8 — proTRAC results folder containing a picture and a FASTA file for each detected macaca cluster. After decompression, the folder can be opened as former session in proTRAC. Alternatively, each file can be opened separately with any standard text-editor or graphic-viewer respectively. [file 1471-2105-13-5-S8.ZIP › proTRAC_results_macaca/bidirectional_clusters/Cluster_43.png]

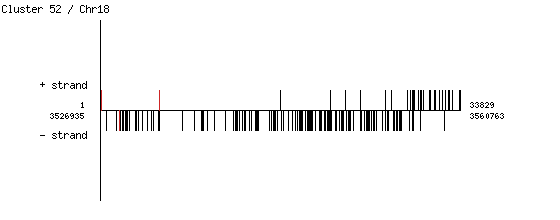

Supplement: Additional file 8 — proTRAC results folder containing a picture and a FASTA file for each detected macaca cluster. After decompression, the folder can be opened as former session in proTRAC. Alternatively, each file can be opened separately with any standard text-editor or graphic-viewer respectively. [file 1471-2105-13-5-S8.ZIP › proTRAC_results_macaca/bidirectional_clusters/Cluster_52.png]

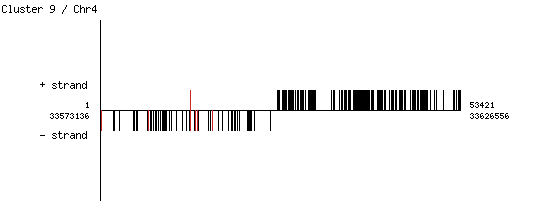

Supplement: Additional file 8 — proTRAC results folder containing a picture and a FASTA file for each detected macaca cluster. After decompression, the folder can be opened as former session in proTRAC. Alternatively, each file can be opened separately with any standard text-editor or graphic-viewer respectively. [file 1471-2105-13-5-S8.ZIP › proTRAC_results_macaca/bidirectional_clusters/Cluster_9.png]

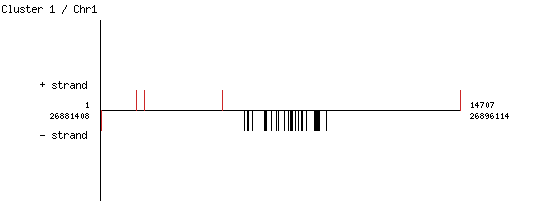

Supplement: Additional file 8 — proTRAC results folder containing a picture and a FASTA file for each detected macaca cluster. After decompression, the folder can be opened as former session in proTRAC. Alternatively, each file can be opened separately with any standard text-editor or graphic-viewer respectively. [file 1471-2105-13-5-S8.ZIP › proTRAC_results_macaca/monodirectional_clusters/Cluster_1.png]

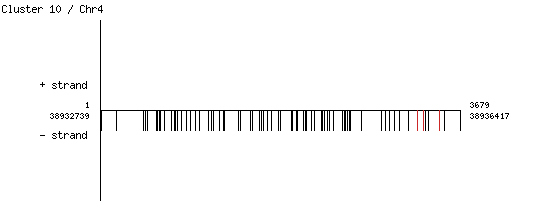

Supplement: Additional file 8 — proTRAC results folder containing a picture and a FASTA file for each detected macaca cluster. After decompression, the folder can be opened as former session in proTRAC. Alternatively, each file can be opened separately with any standard text-editor or graphic-viewer respectively. [file 1471-2105-13-5-S8.ZIP › proTRAC_results_macaca/monodirectional_clusters/Cluster_10.png]

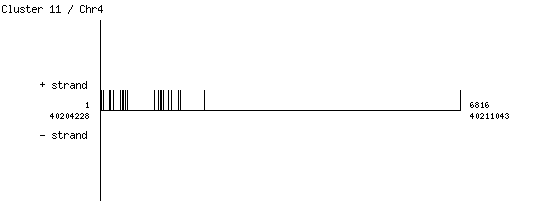

Supplement: Additional file 8 — proTRAC results folder containing a picture and a FASTA file for each detected macaca cluster. After decompression, the folder can be opened as former session in proTRAC. Alternatively, each file can be opened separately with any standard text-editor or graphic-viewer respectively. [file 1471-2105-13-5-S8.ZIP › proTRAC_results_macaca/monodirectional_clusters/Cluster_11.png]

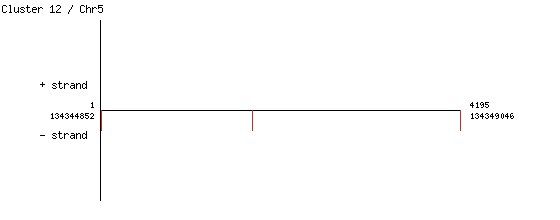

Supplement: Additional file 8 — proTRAC results folder containing a picture and a FASTA file for each detected macaca cluster. After decompression, the folder can be opened as former session in proTRAC. Alternatively, each file can be opened separately with any standard text-editor or graphic-viewer respectively. [file 1471-2105-13-5-S8.ZIP › proTRAC_results_macaca/monodirectional_clusters/Cluster_12.png]

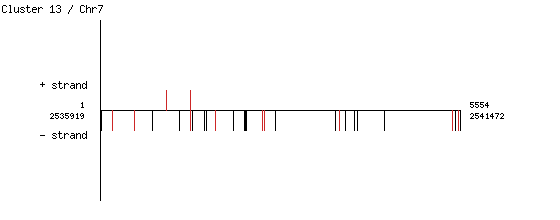

Supplement: Additional file 8 — proTRAC results folder containing a picture and a FASTA file for each detected macaca cluster. After decompression, the folder can be opened as former session in proTRAC. Alternatively, each file can be opened separately with any standard text-editor or graphic-viewer respectively. [file 1471-2105-13-5-S8.ZIP › proTRAC_results_macaca/monodirectional_clusters/Cluster_13.png]

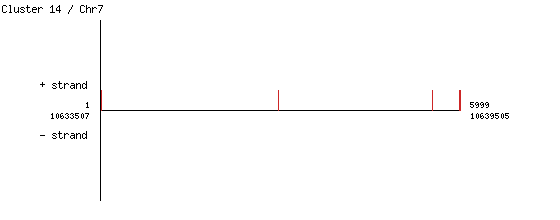

Supplement: Additional file 8 — proTRAC results folder containing a picture and a FASTA file for each detected macaca cluster. After decompression, the folder can be opened as former session in proTRAC. Alternatively, each file can be opened separately with any standard text-editor or graphic-viewer respectively. [file 1471-2105-13-5-S8.ZIP › proTRAC_results_macaca/monodirectional_clusters/Cluster_14.png]

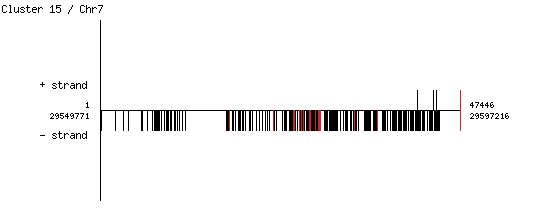

Supplement: Additional file 8 — proTRAC results folder containing a picture and a FASTA file for each detected macaca cluster. After decompression, the folder can be opened as former session in proTRAC. Alternatively, each file can be opened separately with any standard text-editor or graphic-viewer respectively. [file 1471-2105-13-5-S8.ZIP › proTRAC_results_macaca/monodirectional_clusters/Cluster_15.png]

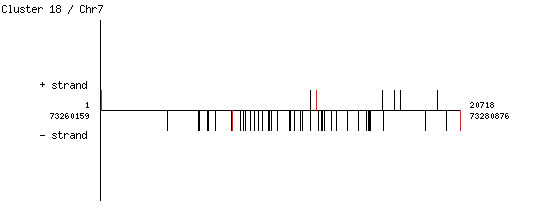

Supplement: Additional file 8 — proTRAC results folder containing a picture and a FASTA file for each detected macaca cluster. After decompression, the folder can be opened as former session in proTRAC. Alternatively, each file can be opened separately with any standard text-editor or graphic-viewer respectively. [file 1471-2105-13-5-S8.ZIP › proTRAC_results_macaca/monodirectional_clusters/Cluster_18.png]

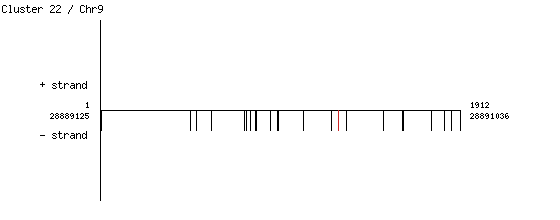

Supplement: Additional file 8 — proTRAC results folder containing a picture and a FASTA file for each detected macaca cluster. After decompression, the folder can be opened as former session in proTRAC. Alternatively, each file can be opened separately with any standard text-editor or graphic-viewer respectively. [file 1471-2105-13-5-S8.ZIP › proTRAC_results_macaca/monodirectional_clusters/Cluster_22.png]

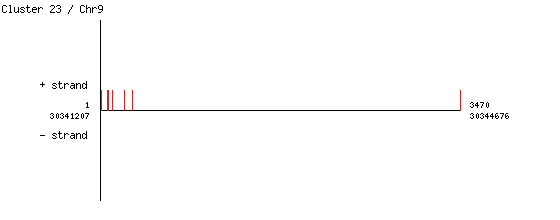

Supplement: Additional file 8 — proTRAC results folder containing a picture and a FASTA file for each detected macaca cluster. After decompression, the folder can be opened as former session in proTRAC. Alternatively, each file can be opened separately with any standard text-editor or graphic-viewer respectively. [file 1471-2105-13-5-S8.ZIP › proTRAC_results_macaca/monodirectional_clusters/Cluster_23.png]

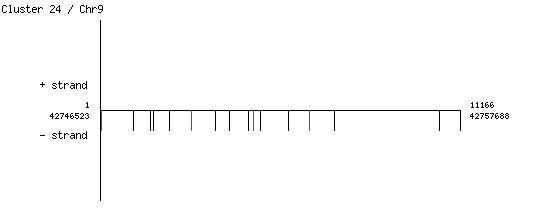

Supplement: Additional file 8 — proTRAC results folder containing a picture and a FASTA file for each detected macaca cluster. After decompression, the folder can be opened as former session in proTRAC. Alternatively, each file can be opened separately with any standard text-editor or graphic-viewer respectively. [file 1471-2105-13-5-S8.ZIP › proTRAC_results_macaca/monodirectional_clusters/Cluster_24.png]

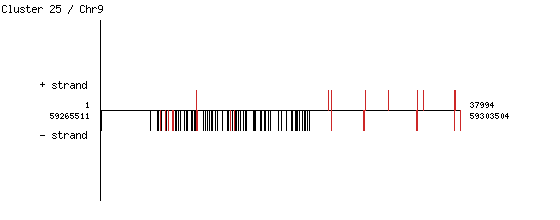

Supplement: Additional file 8 — proTRAC results folder containing a picture and a FASTA file for each detected macaca cluster. After decompression, the folder can be opened as former session in proTRAC. Alternatively, each file can be opened separately with any standard text-editor or graphic-viewer respectively. [file 1471-2105-13-5-S8.ZIP › proTRAC_results_macaca/monodirectional_clusters/Cluster_25.png]

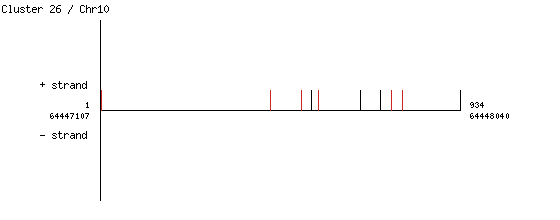

Supplement: Additional file 8 — proTRAC results folder containing a picture and a FASTA file for each detected macaca cluster. After decompression, the folder can be opened as former session in proTRAC. Alternatively, each file can be opened separately with any standard text-editor or graphic-viewer respectively. [file 1471-2105-13-5-S8.ZIP › proTRAC_results_macaca/monodirectional_clusters/Cluster_26.png]

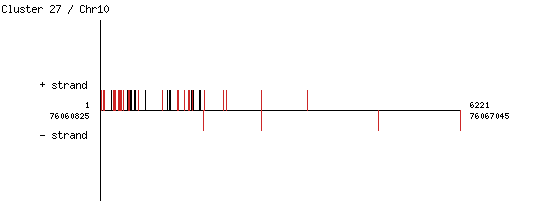

Supplement: Additional file 8 — proTRAC results folder containing a picture and a FASTA file for each detected macaca cluster. After decompression, the folder can be opened as former session in proTRAC. Alternatively, each file can be opened separately with any standard text-editor or graphic-viewer respectively. [file 1471-2105-13-5-S8.ZIP › proTRAC_results_macaca/monodirectional_clusters/Cluster_27.png]

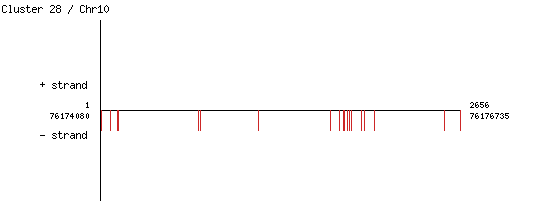

Supplement: Additional file 8 — proTRAC results folder containing a picture and a FASTA file for each detected macaca cluster. After decompression, the folder can be opened as former session in proTRAC. Alternatively, each file can be opened separately with any standard text-editor or graphic-viewer respectively. [file 1471-2105-13-5-S8.ZIP › proTRAC_results_macaca/monodirectional_clusters/Cluster_28.png]

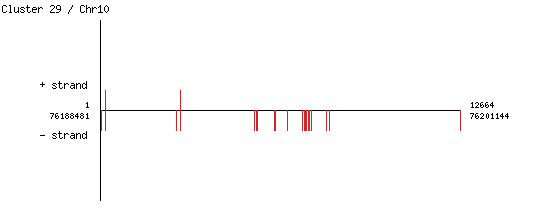

Supplement: Additional file 8 — proTRAC results folder containing a picture and a FASTA file for each detected macaca cluster. After decompression, the folder can be opened as former session in proTRAC. Alternatively, each file can be opened separately with any standard text-editor or graphic-viewer respectively. [file 1471-2105-13-5-S8.ZIP › proTRAC_results_macaca/monodirectional_clusters/Cluster_29.png]

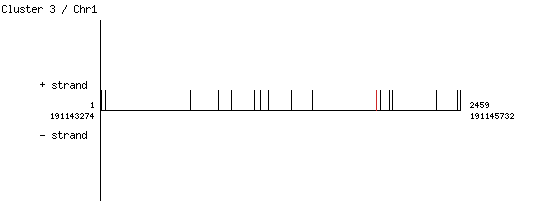

Supplement: Additional file 8 — proTRAC results folder containing a picture and a FASTA file for each detected macaca cluster. After decompression, the folder can be opened as former session in proTRAC. Alternatively, each file can be opened separately with any standard text-editor or graphic-viewer respectively. [file 1471-2105-13-5-S8.ZIP › proTRAC_results_macaca/monodirectional_clusters/Cluster_3.png]

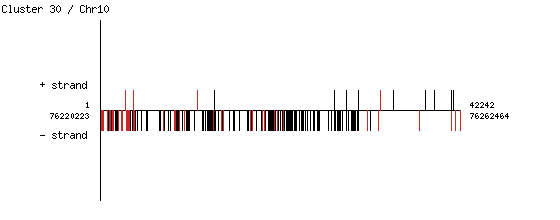

Supplement: Additional file 8 — proTRAC results folder containing a picture and a FASTA file for each detected macaca cluster. After decompression, the folder can be opened as former session in proTRAC. Alternatively, each file can be opened separately with any standard text-editor or graphic-viewer respectively. [file 1471-2105-13-5-S8.ZIP › proTRAC_results_macaca/monodirectional_clusters/Cluster_30.png]

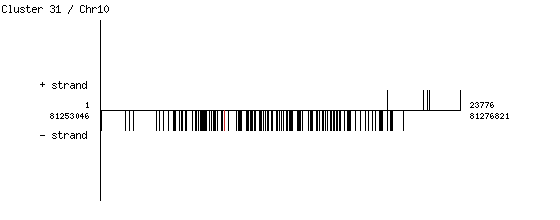

Supplement: Additional file 8 — proTRAC results folder containing a picture and a FASTA file for each detected macaca cluster. After decompression, the folder can be opened as former session in proTRAC. Alternatively, each file can be opened separately with any standard text-editor or graphic-viewer respectively. [file 1471-2105-13-5-S8.ZIP › proTRAC_results_macaca/monodirectional_clusters/Cluster_31.png]

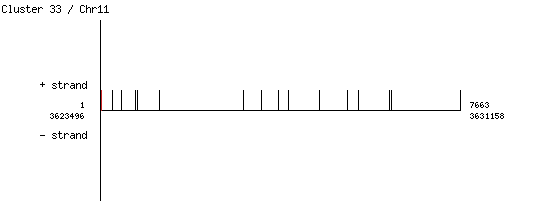

Supplement: Additional file 8 — proTRAC results folder containing a picture and a FASTA file for each detected macaca cluster. After decompression, the folder can be opened as former session in proTRAC. Alternatively, each file can be opened separately with any standard text-editor or graphic-viewer respectively. [file 1471-2105-13-5-S8.ZIP › proTRAC_results_macaca/monodirectional_clusters/Cluster_33.png]

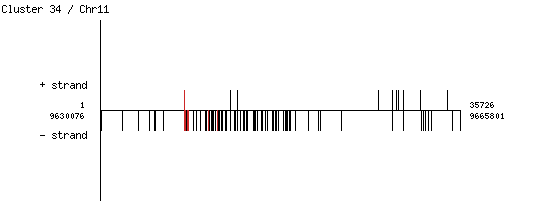

Supplement: Additional file 8 — proTRAC results folder containing a picture and a FASTA file for each detected macaca cluster. After decompression, the folder can be opened as former session in proTRAC. Alternatively, each file can be opened separately with any standard text-editor or graphic-viewer respectively. [file 1471-2105-13-5-S8.ZIP › proTRAC_results_macaca/monodirectional_clusters/Cluster_34.png]

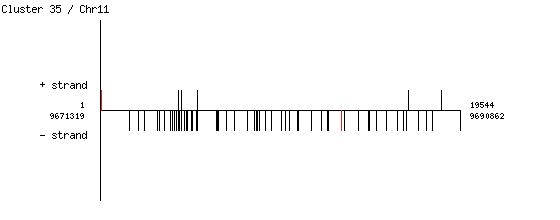

Supplement: Additional file 8 — proTRAC results folder containing a picture and a FASTA file for each detected macaca cluster. After decompression, the folder can be opened as former session in proTRAC. Alternatively, each file can be opened separately with any standard text-editor or graphic-viewer respectively. [file 1471-2105-13-5-S8.ZIP › proTRAC_results_macaca/monodirectional_clusters/Cluster_35.png]

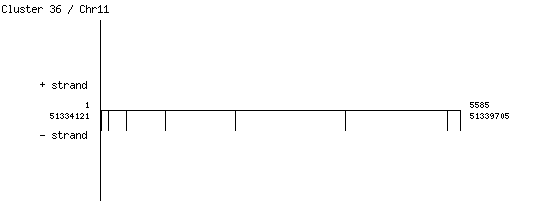

Supplement: Additional file 8 — proTRAC results folder containing a picture and a FASTA file for each detected macaca cluster. After decompression, the folder can be opened as former session in proTRAC. Alternatively, each file can be opened separately with any standard text-editor or graphic-viewer respectively. [file 1471-2105-13-5-S8.ZIP › proTRAC_results_macaca/monodirectional_clusters/Cluster_36.png]

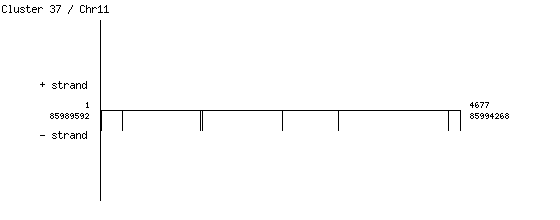

Supplement: Additional file 8 — proTRAC results folder containing a picture and a FASTA file for each detected macaca cluster. After decompression, the folder can be opened as former session in proTRAC. Alternatively, each file can be opened separately with any standard text-editor or graphic-viewer respectively. [file 1471-2105-13-5-S8.ZIP › proTRAC_results_macaca/monodirectional_clusters/Cluster_37.png]

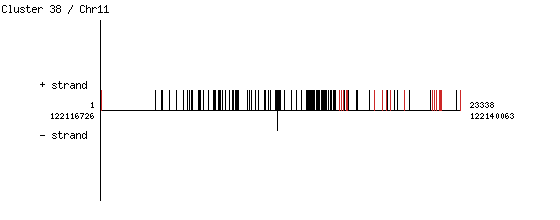

Supplement: Additional file 8 — proTRAC results folder containing a picture and a FASTA file for each detected macaca cluster. After decompression, the folder can be opened as former session in proTRAC. Alternatively, each file can be opened separately with any standard text-editor or graphic-viewer respectively. [file 1471-2105-13-5-S8.ZIP › proTRAC_results_macaca/monodirectional_clusters/Cluster_38.png]

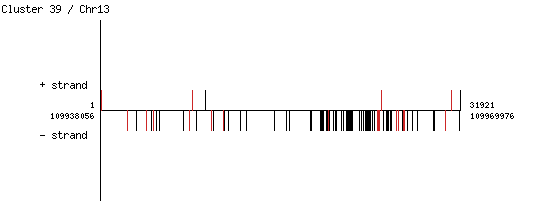

Supplement: Additional file 8 — proTRAC results folder containing a picture and a FASTA file for each detected macaca cluster. After decompression, the folder can be opened as former session in proTRAC. Alternatively, each file can be opened separately with any standard text-editor or graphic-viewer respectively. [file 1471-2105-13-5-S8.ZIP › proTRAC_results_macaca/monodirectional_clusters/Cluster_39.png]

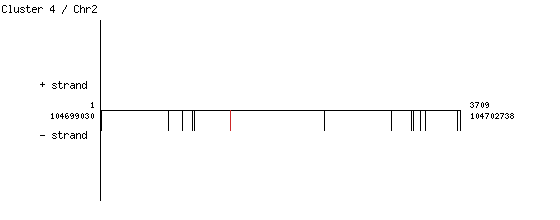

Supplement: Additional file 8 — proTRAC results folder containing a picture and a FASTA file for each detected macaca cluster. After decompression, the folder can be opened as former session in proTRAC. Alternatively, each file can be opened separately with any standard text-editor or graphic-viewer respectively. [file 1471-2105-13-5-S8.ZIP › proTRAC_results_macaca/monodirectional_clusters/Cluster_4.png]

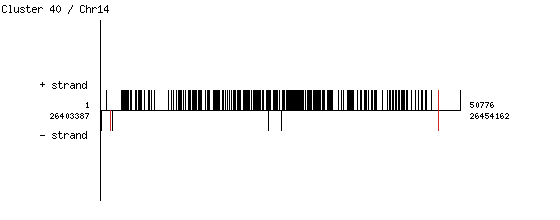

Supplement: Additional file 8 — proTRAC results folder containing a picture and a FASTA file for each detected macaca cluster. After decompression, the folder can be opened as former session in proTRAC. Alternatively, each file can be opened separately with any standard text-editor or graphic-viewer respectively. [file 1471-2105-13-5-S8.ZIP › proTRAC_results_macaca/monodirectional_clusters/Cluster_40.png]

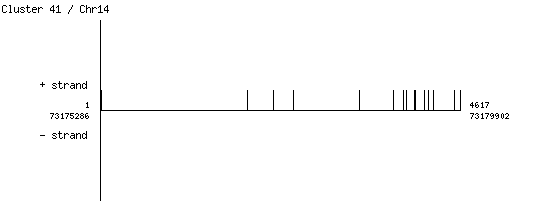

Supplement: Additional file 8 — proTRAC results folder containing a picture and a FASTA file for each detected macaca cluster. After decompression, the folder can be opened as former session in proTRAC. Alternatively, each file can be opened separately with any standard text-editor or graphic-viewer respectively. [file 1471-2105-13-5-S8.ZIP › proTRAC_results_macaca/monodirectional_clusters/Cluster_41.png]

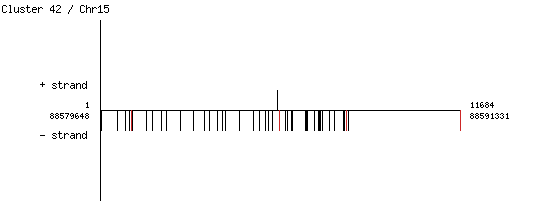

Supplement: Additional file 8 — proTRAC results folder containing a picture and a FASTA file for each detected macaca cluster. After decompression, the folder can be opened as former session in proTRAC. Alternatively, each file can be opened separately with any standard text-editor or graphic-viewer respectively. [file 1471-2105-13-5-S8.ZIP › proTRAC_results_macaca/monodirectional_clusters/Cluster_42.png]

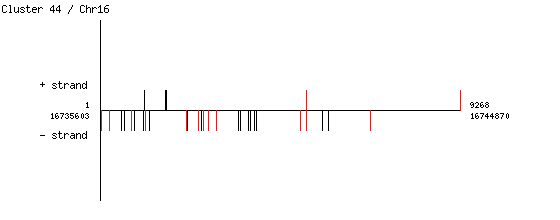

Supplement: Additional file 8 — proTRAC results folder containing a picture and a FASTA file for each detected macaca cluster. After decompression, the folder can be opened as former session in proTRAC. Alternatively, each file can be opened separately with any standard text-editor or graphic-viewer respectively. [file 1471-2105-13-5-S8.ZIP › proTRAC_results_macaca/monodirectional_clusters/Cluster_44.png]

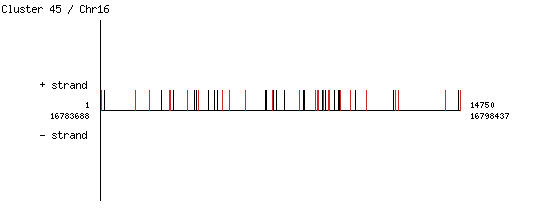

Supplement: Additional file 8 — proTRAC results folder containing a picture and a FASTA file for each detected macaca cluster. After decompression, the folder can be opened as former session in proTRAC. Alternatively, each file can be opened separately with any standard text-editor or graphic-viewer respectively. [file 1471-2105-13-5-S8.ZIP › proTRAC_results_macaca/monodirectional_clusters/Cluster_45.png]

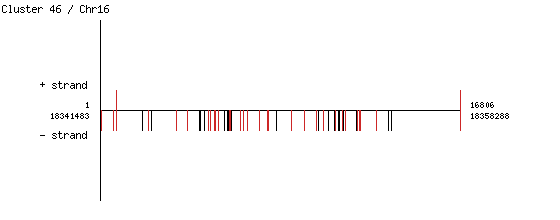

Supplement: Additional file 8 — proTRAC results folder containing a picture and a FASTA file for each detected macaca cluster. After decompression, the folder can be opened as former session in proTRAC. Alternatively, each file can be opened separately with any standard text-editor or graphic-viewer respectively. [file 1471-2105-13-5-S8.ZIP › proTRAC_results_macaca/monodirectional_clusters/Cluster_46.png]

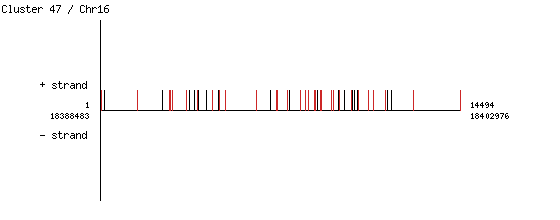

Supplement: Additional file 8 — proTRAC results folder containing a picture and a FASTA file for each detected macaca cluster. After decompression, the folder can be opened as former session in proTRAC. Alternatively, each file can be opened separately with any standard text-editor or graphic-viewer respectively. [file 1471-2105-13-5-S8.ZIP › proTRAC_results_macaca/monodirectional_clusters/Cluster_47.png]

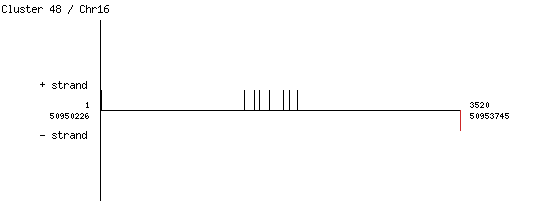

Supplement: Additional file 8 — proTRAC results folder containing a picture and a FASTA file for each detected macaca cluster. After decompression, the folder can be opened as former session in proTRAC. Alternatively, each file can be opened separately with any standard text-editor or graphic-viewer respectively. [file 1471-2105-13-5-S8.ZIP › proTRAC_results_macaca/monodirectional_clusters/Cluster_48.png]

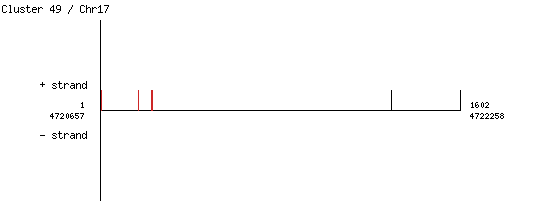

Supplement: Additional file 8 — proTRAC results folder containing a picture and a FASTA file for each detected macaca cluster. After decompression, the folder can be opened as former session in proTRAC. Alternatively, each file can be opened separately with any standard text-editor or graphic-viewer respectively. [file 1471-2105-13-5-S8.ZIP › proTRAC_results_macaca/monodirectional_clusters/Cluster_49.png]

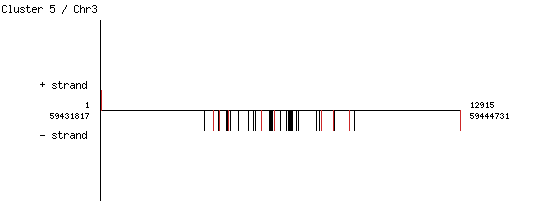

Supplement: Additional file 8 — proTRAC results folder containing a picture and a FASTA file for each detected macaca cluster. After decompression, the folder can be opened as former session in proTRAC. Alternatively, each file can be opened separately with any standard text-editor or graphic-viewer respectively. [file 1471-2105-13-5-S8.ZIP › proTRAC_results_macaca/monodirectional_clusters/Cluster_5.png]

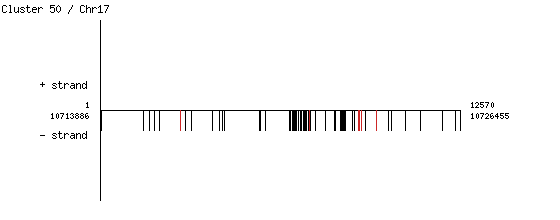

Supplement: Additional file 8 — proTRAC results folder containing a picture and a FASTA file for each detected macaca cluster. After decompression, the folder can be opened as former session in proTRAC. Alternatively, each file can be opened separately with any standard text-editor or graphic-viewer respectively. [file 1471-2105-13-5-S8.ZIP › proTRAC_results_macaca/monodirectional_clusters/Cluster_50.png]

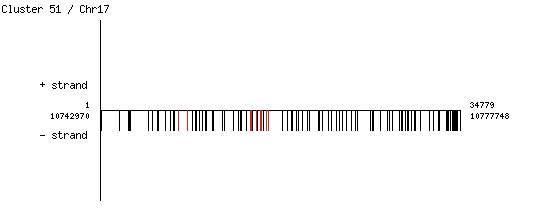

Supplement: Additional file 8 — proTRAC results folder containing a picture and a FASTA file for each detected macaca cluster. After decompression, the folder can be opened as former session in proTRAC. Alternatively, each file can be opened separately with any standard text-editor or graphic-viewer respectively. [file 1471-2105-13-5-S8.ZIP › proTRAC_results_macaca/monodirectional_clusters/Cluster_51.png]

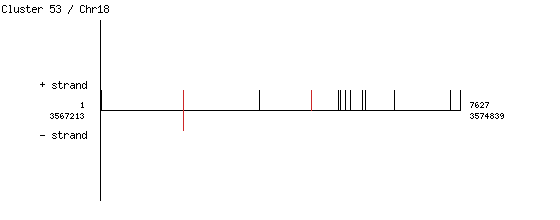

Supplement: Additional file 8 — proTRAC results folder containing a picture and a FASTA file for each detected macaca cluster. After decompression, the folder can be opened as former session in proTRAC. Alternatively, each file can be opened separately with any standard text-editor or graphic-viewer respectively. [file 1471-2105-13-5-S8.ZIP › proTRAC_results_macaca/monodirectional_clusters/Cluster_53.png]

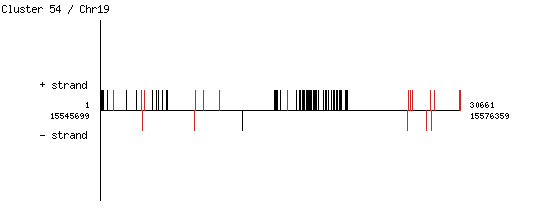

Supplement: Additional file 8 — proTRAC results folder containing a picture and a FASTA file for each detected macaca cluster. After decompression, the folder can be opened as former session in proTRAC. Alternatively, each file can be opened separately with any standard text-editor or graphic-viewer respectively. [file 1471-2105-13-5-S8.ZIP › proTRAC_results_macaca/monodirectional_clusters/Cluster_54.png]

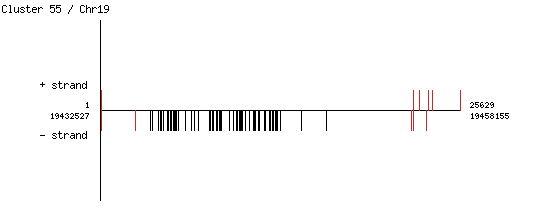

Supplement: Additional file 8 — proTRAC results folder containing a picture and a FASTA file for each detected macaca cluster. After decompression, the folder can be opened as former session in proTRAC. Alternatively, each file can be opened separately with any standard text-editor or graphic-viewer respectively. [file 1471-2105-13-5-S8.ZIP › proTRAC_results_macaca/monodirectional_clusters/Cluster_55.png]

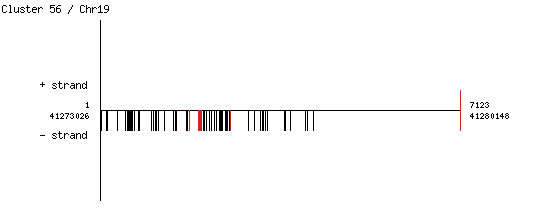

Supplement: Additional file 8 — proTRAC results folder containing a picture and a FASTA file for each detected macaca cluster. After decompression, the folder can be opened as former session in proTRAC. Alternatively, each file can be opened separately with any standard text-editor or graphic-viewer respectively. [file 1471-2105-13-5-S8.ZIP › proTRAC_results_macaca/monodirectional_clusters/Cluster_56.png]

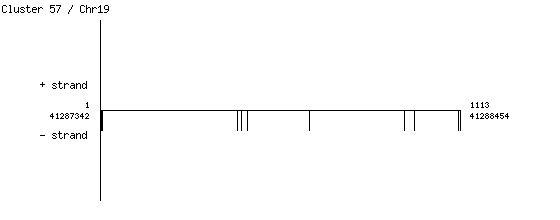

Supplement: Additional file 8 — proTRAC results folder containing a picture and a FASTA file for each detected macaca cluster. After decompression, the folder can be opened as former session in proTRAC. Alternatively, each file can be opened separately with any standard text-editor or graphic-viewer respectively. [file 1471-2105-13-5-S8.ZIP › proTRAC_results_macaca/monodirectional_clusters/Cluster_57.png]

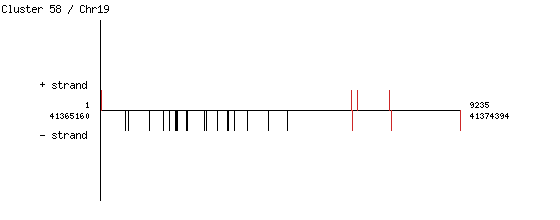

Supplement: Additional file 8 — proTRAC results folder containing a picture and a FASTA file for each detected macaca cluster. After decompression, the folder can be opened as former session in proTRAC. Alternatively, each file can be opened separately with any standard text-editor or graphic-viewer respectively. [file 1471-2105-13-5-S8.ZIP › proTRAC_results_macaca/monodirectional_clusters/Cluster_58.png]

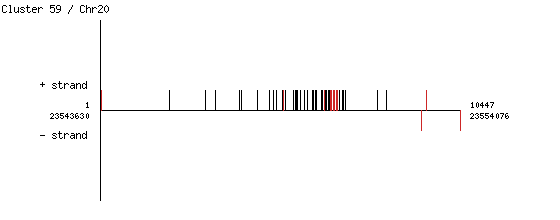

Supplement: Additional file 8 — proTRAC results folder containing a picture and a FASTA file for each detected macaca cluster. After decompression, the folder can be opened as former session in proTRAC. Alternatively, each file can be opened separately with any standard text-editor or graphic-viewer respectively. [file 1471-2105-13-5-S8.ZIP › proTRAC_results_macaca/monodirectional_clusters/Cluster_59.png]

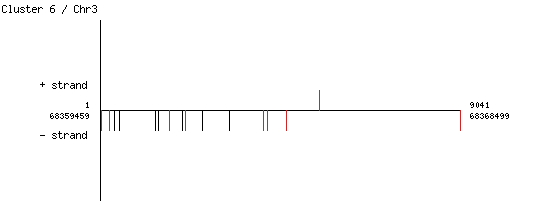

Supplement: Additional file 8 — proTRAC results folder containing a picture and a FASTA file for each detected macaca cluster. After decompression, the folder can be opened as former session in proTRAC. Alternatively, each file can be opened separately with any standard text-editor or graphic-viewer respectively. [file 1471-2105-13-5-S8.ZIP › proTRAC_results_macaca/monodirectional_clusters/Cluster_6.png]

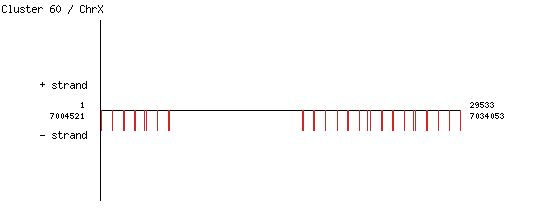

Supplement: Additional file 8 — proTRAC results folder containing a picture and a FASTA file for each detected macaca cluster. After decompression, the folder can be opened as former session in proTRAC. Alternatively, each file can be opened separately with any standard text-editor or graphic-viewer respectively. [file 1471-2105-13-5-S8.ZIP › proTRAC_results_macaca/monodirectional_clusters/Cluster_60.png]

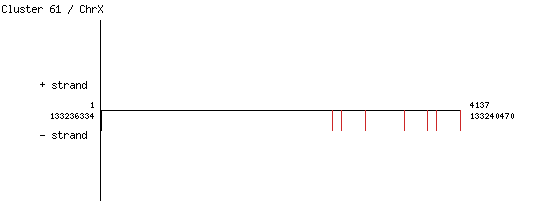

Supplement: Additional file 8 — proTRAC results folder containing a picture and a FASTA file for each detected macaca cluster. After decompression, the folder can be opened as former session in proTRAC. Alternatively, each file can be opened separately with any standard text-editor or graphic-viewer respectively. [file 1471-2105-13-5-S8.ZIP › proTRAC_results_macaca/monodirectional_clusters/Cluster_61.png]

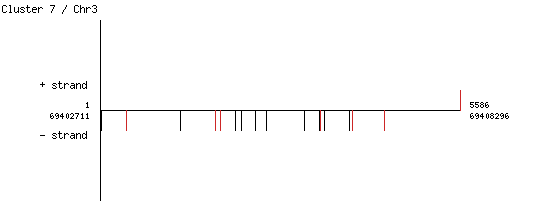

Supplement: Additional file 8 — proTRAC results folder containing a picture and a FASTA file for each detected macaca cluster. After decompression, the folder can be opened as former session in proTRAC. Alternatively, each file can be opened separately with any standard text-editor or graphic-viewer respectively. [file 1471-2105-13-5-S8.ZIP › proTRAC_results_macaca/monodirectional_clusters/Cluster_7.png]

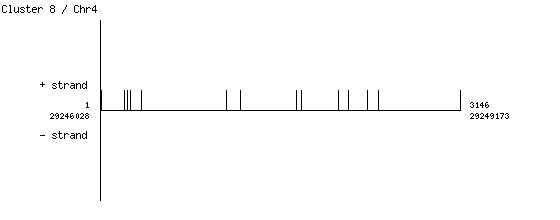

Supplement: Additional file 8 — proTRAC results folder containing a picture and a FASTA file for each detected macaca cluster. After decompression, the folder can be opened as former session in proTRAC. Alternatively, each file can be opened separately with any standard text-editor or graphic-viewer respectively. [file 1471-2105-13-5-S8.ZIP › proTRAC_results_macaca/monodirectional_clusters/Cluster_8.png]

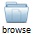

Supplement: Additional file 9 — This folder contains the proTRAC software with all required files and a sample ELAND3 input file. The Perl script (proTRAC.pl) contains the source code of the software that can be run on any platform. Executing Perl scripts requires the installation of a Perl interpreter which is part of a standard Perl distribution like the freely available Strawberry Perl (http://strawberryperl.com/). Perl is preinstalled on most Macintosh and Linux systems. The folder also contains an executable file (proTRAC.exe) which runs on Windows systems without any further requirements. [file 1471-2105-13-5-S9.ZIP › proTRAC/proTRAC_files/browse]

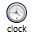

Supplement: Additional file 9 — This folder contains the proTRAC software with all required files and a sample ELAND3 input file. The Perl script (proTRAC.pl) contains the source code of the software that can be run on any platform. Executing Perl scripts requires the installation of a Perl interpreter which is part of a standard Perl distribution like the freely available Strawberry Perl (http://strawberryperl.com/). Perl is preinstalled on most Macintosh and Linux systems. The folder also contains an executable file (proTRAC.exe) which runs on Windows systems without any further requirements. [file 1471-2105-13-5-S9.ZIP › proTRAC/proTRAC_files/clock]

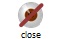

Supplement: Additional file 9 — This folder contains the proTRAC software with all required files and a sample ELAND3 input file. The Perl script (proTRAC.pl) contains the source code of the software that can be run on any platform. Executing Perl scripts requires the installation of a Perl interpreter which is part of a standard Perl distribution like the freely available Strawberry Perl (http://strawberryperl.com/). Perl is preinstalled on most Macintosh and Linux systems. The folder also contains an executable file (proTRAC.exe) which runs on Windows systems without any further requirements. [file 1471-2105-13-5-S9.ZIP › proTRAC/proTRAC_files/closeses]

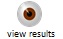

Supplement: Additional file 9 — This folder contains the proTRAC software with all required files and a sample ELAND3 input file. The Perl script (proTRAC.pl) contains the source code of the software that can be run on any platform. Executing Perl scripts requires the installation of a Perl interpreter which is part of a standard Perl distribution like the freely available Strawberry Perl (http://strawberryperl.com/). Perl is preinstalled on most Macintosh and Linux systems. The folder also contains an executable file (proTRAC.exe) which runs on Windows systems without any further requirements. [file 1471-2105-13-5-S9.ZIP › proTRAC/proTRAC_files/cses]

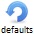

Supplement: Additional file 9 — This folder contains the proTRAC software with all required files and a sample ELAND3 input file. The Perl script (proTRAC.pl) contains the source code of the software that can be run on any platform. Executing Perl scripts requires the installation of a Perl interpreter which is part of a standard Perl distribution like the freely available Strawberry Perl (http://strawberryperl.com/). Perl is preinstalled on most Macintosh and Linux systems. The folder also contains an executable file (proTRAC.exe) which runs on Windows systems without any further requirements. [file 1471-2105-13-5-S9.ZIP › proTRAC/proTRAC_files/defaults]

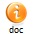

Supplement: Additional file 9 — This folder contains the proTRAC software with all required files and a sample ELAND3 input file. The Perl script (proTRAC.pl) contains the source code of the software that can be run on any platform. Executing Perl scripts requires the installation of a Perl interpreter which is part of a standard Perl distribution like the freely available Strawberry Perl (http://strawberryperl.com/). Perl is preinstalled on most Macintosh and Linux systems. The folder also contains an executable file (proTRAC.exe) which runs on Windows systems without any further requirements. [file 1471-2105-13-5-S9.ZIP › proTRAC/proTRAC_files/doc]

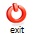

Supplement: Additional file 9 — This folder contains the proTRAC software with all required files and a sample ELAND3 input file. The Perl script (proTRAC.pl) contains the source code of the software that can be run on any platform. Executing Perl scripts requires the installation of a Perl interpreter which is part of a standard Perl distribution like the freely available Strawberry Perl (http://strawberryperl.com/). Perl is preinstalled on most Macintosh and Linux systems. The folder also contains an executable file (proTRAC.exe) which runs on Windows systems without any further requirements. [file 1471-2105-13-5-S9.ZIP › proTRAC/proTRAC_files/exit]

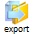

Supplement: Additional file 9 — This folder contains the proTRAC software with all required files and a sample ELAND3 input file. The Perl script (proTRAC.pl) contains the source code of the software that can be run on any platform. Executing Perl scripts requires the installation of a Perl interpreter which is part of a standard Perl distribution like the freely available Strawberry Perl (http://strawberryperl.com/). Perl is preinstalled on most Macintosh and Linux systems. The folder also contains an executable file (proTRAC.exe) which runs on Windows systems without any further requirements. [file 1471-2105-13-5-S9.ZIP › proTRAC/proTRAC_files/export]

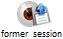

Supplement: Additional file 9 — This folder contains the proTRAC software with all required files and a sample ELAND3 input file. The Perl script (proTRAC.pl) contains the source code of the software that can be run on any platform. Executing Perl scripts requires the installation of a Perl interpreter which is part of a standard Perl distribution like the freely available Strawberry Perl (http://strawberryperl.com/). Perl is preinstalled on most Macintosh and Linux systems. The folder also contains an executable file (proTRAC.exe) which runs on Windows systems without any further requirements. [file 1471-2105-13-5-S9.ZIP › proTRAC/proTRAC_files/fses]

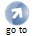

Supplement: Additional file 9 — This folder contains the proTRAC software with all required files and a sample ELAND3 input file. The Perl script (proTRAC.pl) contains the source code of the software that can be run on any platform. Executing Perl scripts requires the installation of a Perl interpreter which is part of a standard Perl distribution like the freely available Strawberry Perl (http://strawberryperl.com/). Perl is preinstalled on most Macintosh and Linux systems. The folder also contains an executable file (proTRAC.exe) which runs on Windows systems without any further requirements. [file 1471-2105-13-5-S9.ZIP › proTRAC/proTRAC_files/goto]

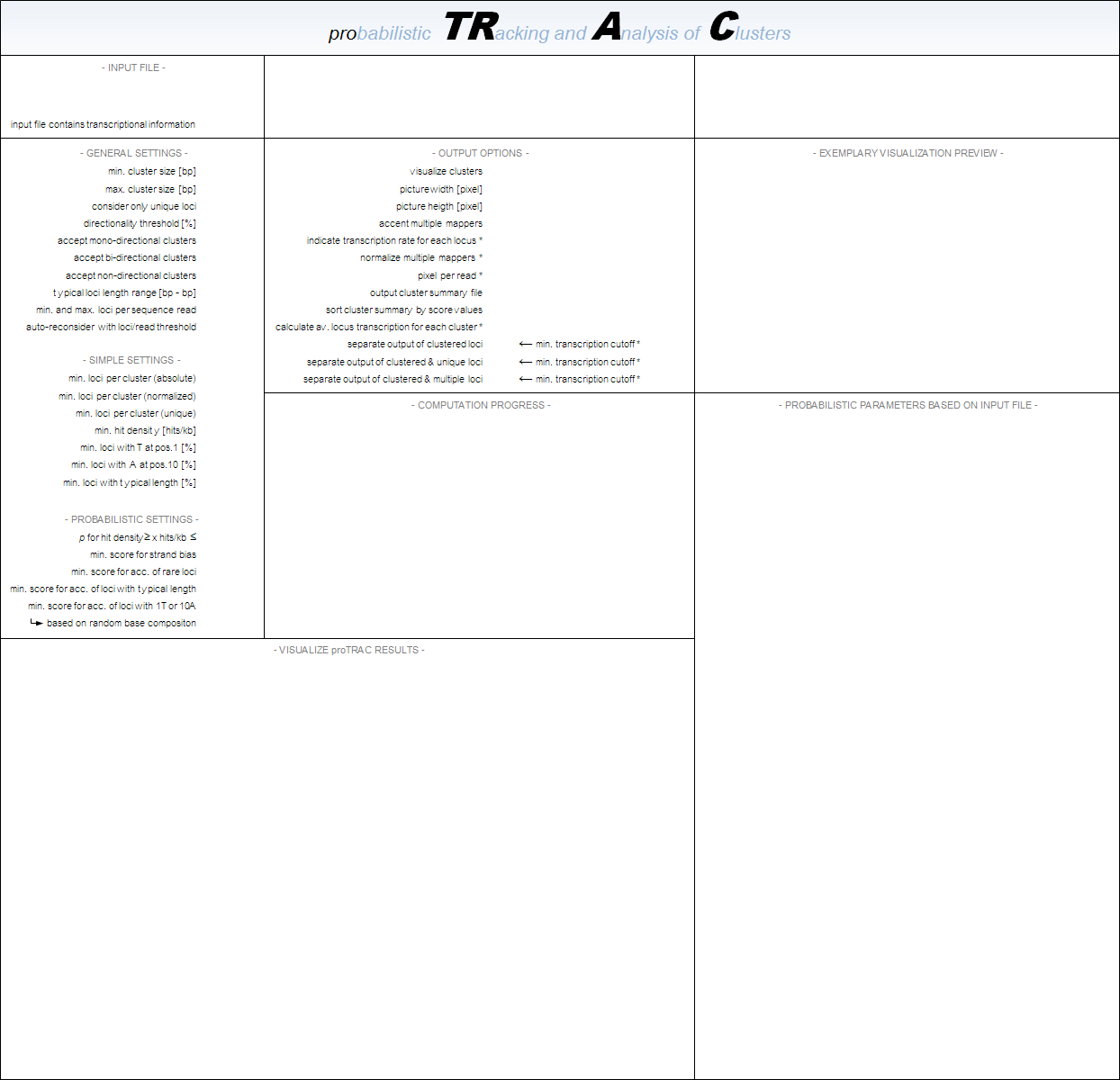

Supplement: Additional file 9 — This folder contains the proTRAC software with all required files and a sample ELAND3 input file. The Perl script (proTRAC.pl) contains the source code of the software that can be run on any platform. Executing Perl scripts requires the installation of a Perl interpreter which is part of a standard Perl distribution like the freely available Strawberry Perl (http://strawberryperl.com/). Perl is preinstalled on most Macintosh and Linux systems. The folder also contains an executable file (proTRAC.exe) which runs on Windows systems without any further requirements. [file 1471-2105-13-5-S9.ZIP › proTRAC/proTRAC_files/gui]

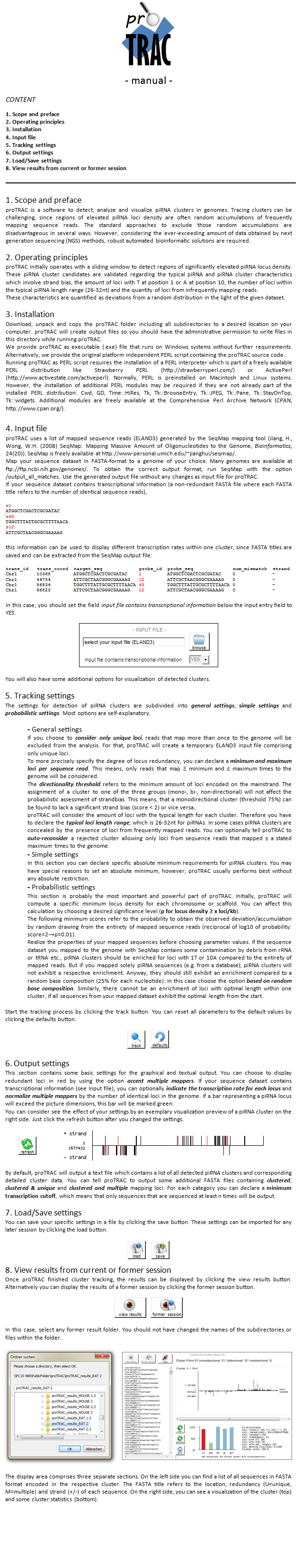

Supplement: Additional file 9 — This folder contains the proTRAC software with all required files and a sample ELAND3 input file. The Perl script (proTRAC.pl) contains the source code of the software that can be run on any platform. Executing Perl scripts requires the installation of a Perl interpreter which is part of a standard Perl distribution like the freely available Strawberry Perl (http://strawberryperl.com/). Perl is preinstalled on most Macintosh and Linux systems. The folder also contains an executable file (proTRAC.exe) which runs on Windows systems without any further requirements. [file 1471-2105-13-5-S9.ZIP › proTRAC/proTRAC_files/info]

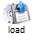

Supplement: Additional file 9 — This folder contains the proTRAC software with all required files and a sample ELAND3 input file. The Perl script (proTRAC.pl) contains the source code of the software that can be run on any platform. Executing Perl scripts requires the installation of a Perl interpreter which is part of a standard Perl distribution like the freely available Strawberry Perl (http://strawberryperl.com/). Perl is preinstalled on most Macintosh and Linux systems. The folder also contains an executable file (proTRAC.exe) which runs on Windows systems without any further requirements. [file 1471-2105-13-5-S9.ZIP › proTRAC/proTRAC_files/load]

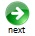

Supplement: Additional file 9 — This folder contains the proTRAC software with all required files and a sample ELAND3 input file. The Perl script (proTRAC.pl) contains the source code of the software that can be run on any platform. Executing Perl scripts requires the installation of a Perl interpreter which is part of a standard Perl distribution like the freely available Strawberry Perl (http://strawberryperl.com/). Perl is preinstalled on most Macintosh and Linux systems. The folder also contains an executable file (proTRAC.exe) which runs on Windows systems without any further requirements. [file 1471-2105-13-5-S9.ZIP › proTRAC/proTRAC_files/next]

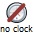

Supplement: Additional file 9 — This folder contains the proTRAC software with all required files and a sample ELAND3 input file. The Perl script (proTRAC.pl) contains the source code of the software that can be run on any platform. Executing Perl scripts requires the installation of a Perl interpreter which is part of a standard Perl distribution like the freely available Strawberry Perl (http://strawberryperl.com/). Perl is preinstalled on most Macintosh and Linux systems. The folder also contains an executable file (proTRAC.exe) which runs on Windows systems without any further requirements. [file 1471-2105-13-5-S9.ZIP › proTRAC/proTRAC_files/noclock]

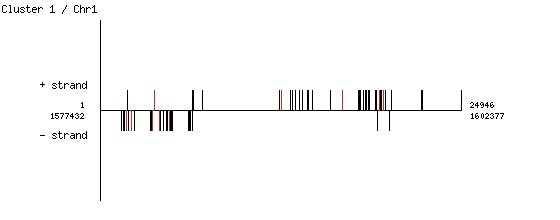

Supplement: Additional file 9 — This folder contains the proTRAC software with all required files and a sample ELAND3 input file. The Perl script (proTRAC.pl) contains the source code of the software that can be run on any platform. Executing Perl scripts requires the installation of a Perl interpreter which is part of a standard Perl distribution like the freely available Strawberry Perl (http://strawberryperl.com/). Perl is preinstalled on most Macintosh and Linux systems. The folder also contains an executable file (proTRAC.exe) which runs on Windows systems without any further requirements. [file 1471-2105-13-5-S9.ZIP › proTRAC/proTRAC_files/prev_temp]

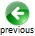

Supplement: Additional file 9 — This folder contains the proTRAC software with all required files and a sample ELAND3 input file. The Perl script (proTRAC.pl) contains the source code of the software that can be run on any platform. Executing Perl scripts requires the installation of a Perl interpreter which is part of a standard Perl distribution like the freely available Strawberry Perl (http://strawberryperl.com/). Perl is preinstalled on most Macintosh and Linux systems. The folder also contains an executable file (proTRAC.exe) which runs on Windows systems without any further requirements. [file 1471-2105-13-5-S9.ZIP › proTRAC/proTRAC_files/previous]

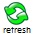

Supplement: Additional file 9 — This folder contains the proTRAC software with all required files and a sample ELAND3 input file. The Perl script (proTRAC.pl) contains the source code of the software that can be run on any platform. Executing Perl scripts requires the installation of a Perl interpreter which is part of a standard Perl distribution like the freely available Strawberry Perl (http://strawberryperl.com/). Perl is preinstalled on most Macintosh and Linux systems. The folder also contains an executable file (proTRAC.exe) which runs on Windows systems without any further requirements. [file 1471-2105-13-5-S9.ZIP › proTRAC/proTRAC_files/refresh]

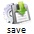

Supplement: Additional file 9 — This folder contains the proTRAC software with all required files and a sample ELAND3 input file. The Perl script (proTRAC.pl) contains the source code of the software that can be run on any platform. Executing Perl scripts requires the installation of a Perl interpreter which is part of a standard Perl distribution like the freely available Strawberry Perl (http://strawberryperl.com/). Perl is preinstalled on most Macintosh and Linux systems. The folder also contains an executable file (proTRAC.exe) which runs on Windows systems without any further requirements. [file 1471-2105-13-5-S9.ZIP › proTRAC/proTRAC_files/save]

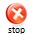

Supplement: Additional file 9 — This folder contains the proTRAC software with all required files and a sample ELAND3 input file. The Perl script (proTRAC.pl) contains the source code of the software that can be run on any platform. Executing Perl scripts requires the installation of a Perl interpreter which is part of a standard Perl distribution like the freely available Strawberry Perl (http://strawberryperl.com/). Perl is preinstalled on most Macintosh and Linux systems. The folder also contains an executable file (proTRAC.exe) which runs on Windows systems without any further requirements. [file 1471-2105-13-5-S9.ZIP › proTRAC/proTRAC_files/stop]

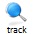

Supplement: Additional file 9 — This folder contains the proTRAC software with all required files and a sample ELAND3 input file. The Perl script (proTRAC.pl) contains the source code of the software that can be run on any platform. Executing Perl scripts requires the installation of a Perl interpreter which is part of a standard Perl distribution like the freely available Strawberry Perl (http://strawberryperl.com/). Perl is preinstalled on most Macintosh and Linux systems. The folder also contains an executable file (proTRAC.exe) which runs on Windows systems without any further requirements. [file 1471-2105-13-5-S9.ZIP › proTRAC/proTRAC_files/track]
