# Supplementary material for: proTRAC - a software for probabilistic piRNA cluster detection, visualization and analysis
Source: BMC Bioinformatics. 2012 Jan 10;13:5. doi: 10.1186/1471-2105-13-5 (PMC3293768; doi:10.1186/1471-2105-13-5)
Supplement: Additional file 9 — This folder contains the proTRAC software with all required files and a sample ELAND3 input file. The Perl script (proTRAC.pl) contains the source code of the software that can be run on any platform. Executing Perl scripts requires the installation of a Perl interpreter which is part of a standard Perl distribution like the freely available Strawberry Perl (http://strawberryperl.com/). Perl is preinstalled on most Macintosh and Linux systems. The folder also contains an executable file (proTRAC.exe) which runs on Windows systems without any further requirements. [file 1471-2105-13-5-S9.ZIP › proTRAC/proTRAC_documentation.pdf]

# - manual -

CONTENT

1. Scope and preface
2. Operating principles
3. Installation
4. Input file
5. Tracking settings
6. Output settings
7. Load/Save settings
8. View results from current or former session

1. Scope and preface

proTRAC is a software to detect, analyze and visualize piRNA clusters in genomes. Tracing clusters can be challenging, since regions of elevated piRNA loci density are often random accumulations of frequently mapping sequence reads. The standard approaches to exclude those random accumulations are disadvantageous in several ways. However, considering the ever-exceeding amount of data obtained by next generation sequencing (NGS) methods, robust automated bioinformatic solutions are required.

2. Operating principles

proTRAC initially operates with a sliding window to detect regions of significantly elevated piRNA locus density. These piRNA cluster candidates are validated regarding the typical piRNA and piRNA cluster characteristics which involve strand bias, the amount of loci with T at position 1 or A at position 10, the number of loci within the typical piRNA length range (26-32nt) and the quantity of loci from infrequently mapping reads.
These characteristics are quantified as deviations from a random distribution in the light of the given dataset.

3. Installation

Download, unpack and copy the proTRAC folder including all subdirectories to a desired location on your computer. proTRAC will create output files so you should have the administrative permission to write files in this directory while running proTRAC.
We provide proTRAC as executable (.exe) file that runs on Windows systems without further requirements. Alternatively, we provide the original platform independent PERL script containing the proTRAC source code.
Running proTRAC as PERL script requires the installation of a PERL interpreter which is part of a freely available PERL distribution like Strawberry PERL (http://strawberryperl.com/) or ActivePerl (http://www.activestate.com/activeperl). Normally, PERL is preinstalled on Macintosh and Linux systems. However, the installation of additional PERL modules may be required if they are not already part of the installed PERL distribution: Cwd, GD, Time::HiRes, Tk, Tk::BrowseEntry, Tk::JPEG, Tk::Pane, Tk::StayOnTop, Tk::widgets. Additional modules are freely available at the Comprehensive Perl Archive Network (CPAN, http://www.cpan.org/).

4. Input file

proTRAC uses a list of mapped sequence reads (ELAND3) generated by the SeqMap mapping tool (Jiang, H., Wong, W.H. (2008) SeqMap: Mapping Massive Amount of Oligonucleotides to the Genome, *Bioinformatics,* 24(20)). SeqMap is freely available at http://www-personal.umich.edu/~jianghui/seqmap/.
Map your sequence dataset in FASTA-format to a genome of your choice. Many genomes are available at ftp://ftp.ncbi.nih.gov/genomes/. To obtain the correct output format, run SeqMap with the option /output_all_matches. Use the generated output file without any changes as input file for proTRAC.
If your sequence dataset contains transcriptional information (a non-redundant FASTA file where each FASTA title refers to the number of identical sequence reads),

```
>1
ATGGCTCGACTCGCGATAC
>45
TGGCTTTATTGCGCTTTTAACA
>12
ATTCGCTAACGGGCGAAAAG
```

this information can be used to display different transcription rates within one cluster, since FASTA titles are saved and can be extracted from the SeqMap output file:

trans_id	trans_coord	target_seq	probe_id	probe_seq	num_mismatch	strand
Chr1	10368	ATGGCTCGACTCGCGATAC	1	ATGGCTCGACTCGCGATAC	0	-
Chr1	44754	ATTCGCTAACGGGCGAAAAG	12	ATTCGCTAACGGGCGAAAAG	0	-
Chr1	56834	TGGCTTTATTGCGCTTTTAACA	45	TGGCTTTATTGCGCTTTTAACA	0	-
Chr1	96823	ATTCGCTAACGGGCGAAAAG	12	ATTCGCTAACGGGCGAAAAG	0	-

In this case, you should set the field *input file contains transcriptional information* below the input entry field to *YES*.

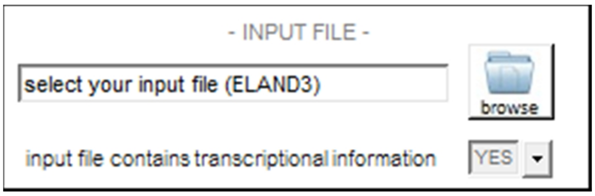

You will also have some additional options for visualization of detected clusters.

5. Tracking settings

The settings for detection of piRNA clusters are subdivided into *general settings*, *simple settings* and *probabilistic settings*. Most options are self-explanatory.

- General settings

If you choose to *consider only unique loci*, reads that map more than once to the genome will be excluded from the analysis. For that, proTRAC will create a temporary ELAND3 input file comprising only unique loci.
To more precisely specify the degree of locus redundancy, you can declare a *minimum and maximum loci per sequence read*. This means, only reads that map ≥ minimum and ≤ maximum times to the genome will be considered.
The *directionality threshold* refers to the minimum amount of loci encoded on the mainstrand. The assignment of a cluster to one of the three groups (mono-, bi-, non-directional) will not affect the probabilistic assessment of strandbias. This means, that a monodirectional cluster (threshold 75%) can be found to lack a significant strand bias (score < 2) or vice versa.
proTRAC will consider the amount of loci with the typical length for each cluster. Therefore you have to declare the *typical loci length range*, which is 26-32nt for piRNAs. In some cases piRNA clusters are concealed by the presence of loci from frequently mapped reads. You can optionally tell proTRAC to *auto-reconsider* a rejected cluster allowing only loci from sequence reads that mapped ≤ a stated maximum times to the genome.

- Simple settings

In this section you can declare specific absolute minimum requirements for piRNA clusters. You may have special reasons to set an absolute minimum, however, proTRAC usually performs best without any absolute restriction.

- Probabilistic settings

This section is probably the most important and powerful part of proTRAC. Initially, proTRAC will compute a specific minimum locus density for each chromosome or scaffold. You can affect this calculation by choosing a desired significance level (p for locus density ≥ x loci/kb).

The following minimum scores refer to the probability to obtain the observed deviation/accumulation by random drawing from the entirety of mapped sequence reads (reciprocal of log10 of probability: score=2→p=0.01).

Realize the properties of your mapped sequences before choosing parameter values. If the sequence dataset you mapped to the genome with SeqMap contains some contamination by debris from rRNA or tRNA etc., piRNA clusters should be enriched for loci with 1T or 10A compared to the entirety of mapped reads. But if you mapped solely piRNA sequences (e.g. from a database), piRNA clusters will not exhibit a respective enrichment. Anyway, they should still exhibit an enrichment compared to a random base composition (25% for each nucleotide). In this case choose the option *based on random base composition*. Similarly, there cannot be an enrichment of loci with optimal length within one cluster, if all sequences from your mapped dataset exhibit the optimal length from the start.

Start the tracking process by clicking the track button. You can reset all parameters to the default values by clicking the defaults button.

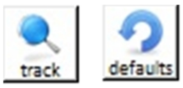

6. Output settings

This section contains some basic settings for the graphical and textual output. You can choose to display redundant loci in red by using the option *accent multiple mappers*. If your sequence dataset contains transcriptional information (see Input file), you can optionally *indicate the transcription rate for each locus* and *normalize multiple mappers* by the number of identical loci in the genome. If a bar representing a piRNA locus will exceed the picture dimensions, this bar will be marked green.

You can consider see the effect of your settings by an exemplary visualization preview of a piRNA cluster on the right side. Just click the refresh button after you changed the settings.

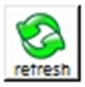

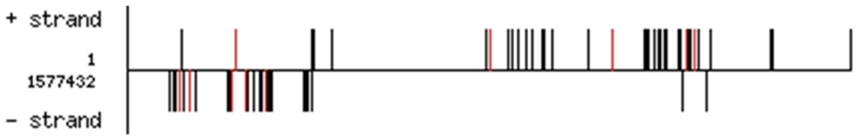

By default, proTRAC will output a text file which contains a list of all detected piRNA clusters and corresponding detailed cluster data. You can tell proTRAC to output some additional FASTA files containing *clustered*, *clustered & unique* and *clustered and multiple* mapping loci. For each category you can declare a minimum transcription cutoff, which means that only sequences that are sequenced at least n times will be output.

7. Load/Save settings

You can save your specific settings in a file by clicking the save button. These settings can be imported for any later session by clicking the load button.

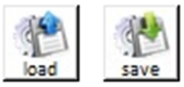

8. View results from current or former session

Once proTRAC finished cluster tracking, the results can be displayed by clicking the view results button. Alternatively you can display the results of a former session by clicking the former session button.

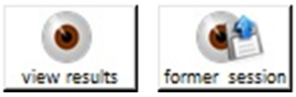

In this case, select any former result folder. You should not have changed the names of the subdirectories or files within the folder.

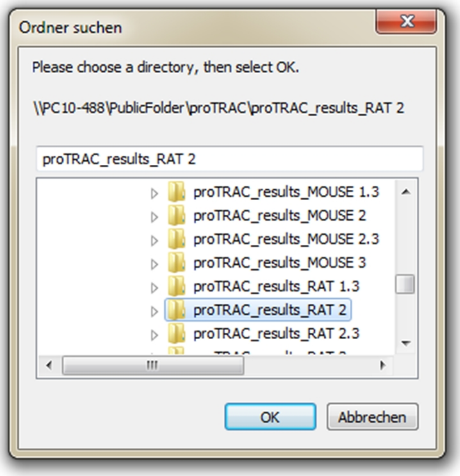

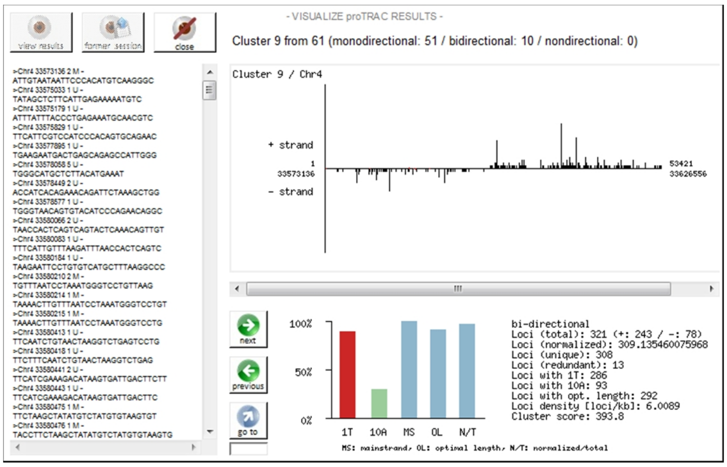

The display area comprises three separate sections. On the left side you can find a list of all sequences in FASTA format encoded in the respective cluster. The FASTA title refers to the location, redundancy (U=unique, M=multiple) and strand (+/-) of each sequence. On the right side, you can see a visualization of the cluster (top) and some cluster statistics (bottom).
